# Supplementary material for: Citrobacter rodentium Subverts ATP Flux and Cholesterol Homeostasis in Intestinal Epithelial Cells In Vivo
Source: Cell Metab. 2017 Nov 7;26(5):738–752.e6. doi: 10.1016/j.cmet.2017.09.003 (PMC5695859; doi:10.1016/j.cmet.2017.09.003)
Supplement: Document S1. Figures S1–S4 and Tables S1–S3 [file mmc1.pdf]

Cell Metabolism, Volume *26*

Supplemental Information

Citrobacter rodentium Subverts ATP Flux and Cholesterol Homeostasis in Intestinal Epithelial Cells *In Vivo*

Cedric N. Berger, Valerie F. Crepin, Theodoros I. Roumeliotis, James C. Wright, Danielle Carson, Meirav Pevsner-Fischer, R. Christopher D. Furniss, Gordon Dougan, Mally Dori-Bachash, Lu Yu, Abigail Clements, James W. Collins, Eran Elinav, Gerald J. Larrouy-Maumus, Jyoti S. Choudhary, and Gad Frankel

Supplementary figure and tables

Figure S1. Relative abundance of metabolic proteins (related to Figure 2A, 2D, 3A, 5A, 5B, 6A and 6C).

Bar plots showing the relative abundance of the individual proteins: **A.** mitochondrial membrane proteins (related to Figure 2A), **B.** TCA cycle (related to Figure 2D), **C.** cardiolipin biosynthesis (related to Figure 3A), **D.** Glycolysis (related to Figure 5A), **E.** creatine biosynthesis (related to Figure 5B), **F.** AMPK pathway (related to Figure 6A) and **G.** cholesterol biosynthesis (related to Figure 6C). Proteins below the significant value (log2FC >0.59 or <-0.59) are shown in a lighter shade.

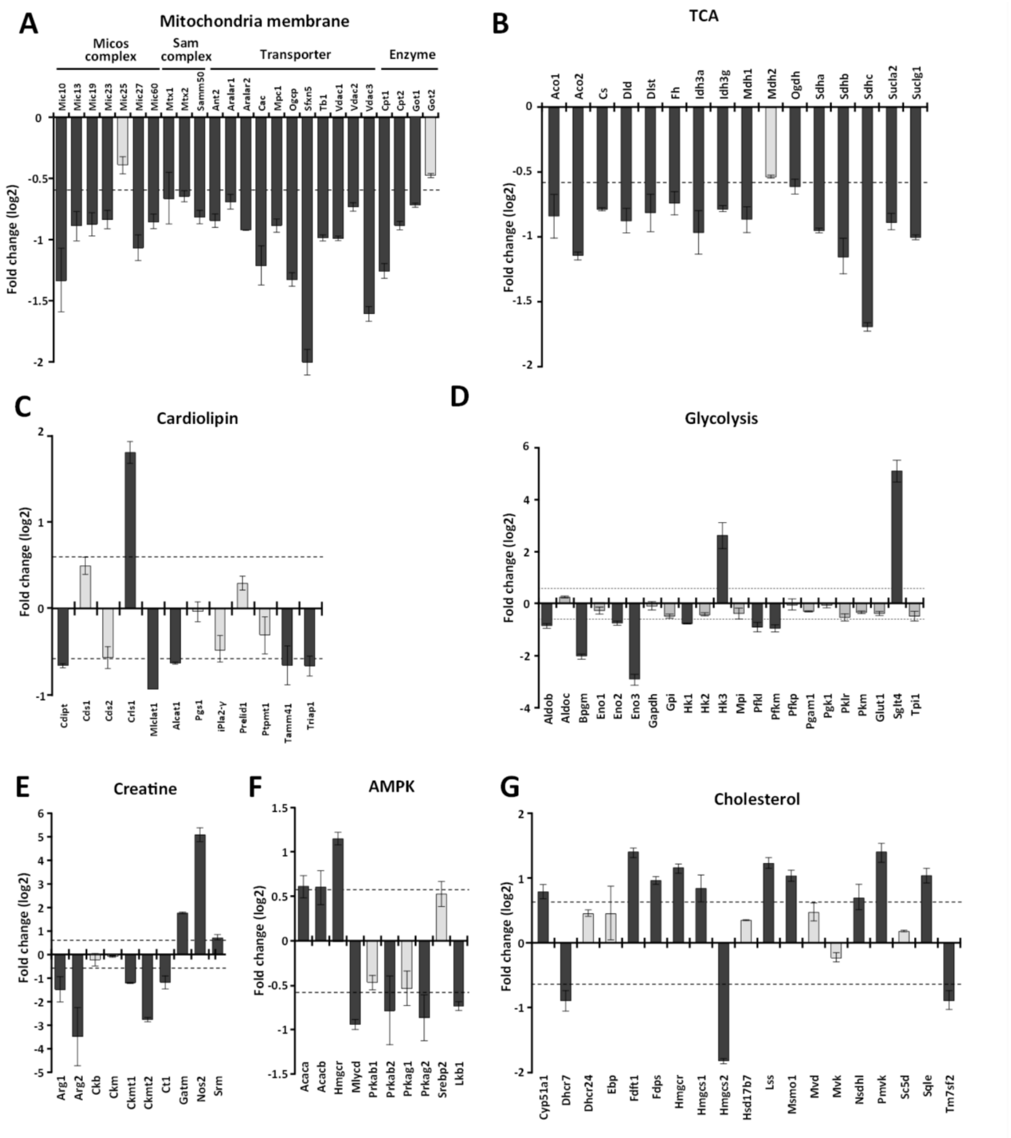

Figure S1

Figure S2. ***C. rodentium*** **impacts β-oxidation (related to Figure 2A).**

Schematic representation of the β-oxidation cycle, with the affected proteins during infection (related to Figure 2A). The bar plot shows the relative abundances of the individual proteins. Proteins below the significant value (log2FC >0.59 or <-0.59) are shown in a lighter shade.

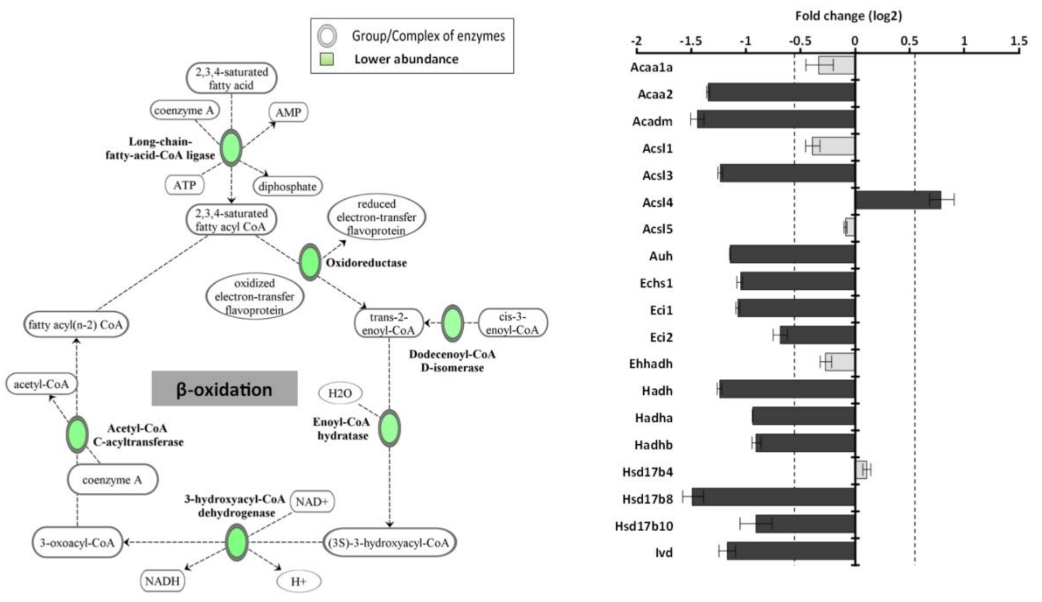

Figure S2

Figure S3. ***C. rodentium*** **impacts lipid elongation (related to Figure 3B).**

Schematic representation of lipid elongation and phosphatidic acid production, with the affected proteins during infection (related to Figure 3B). The bar plot shows the relative abundances of the individual enzymes. Proteins below the significant value (log2FC>0.59 or <-0.59) are shown in a lighter shade.

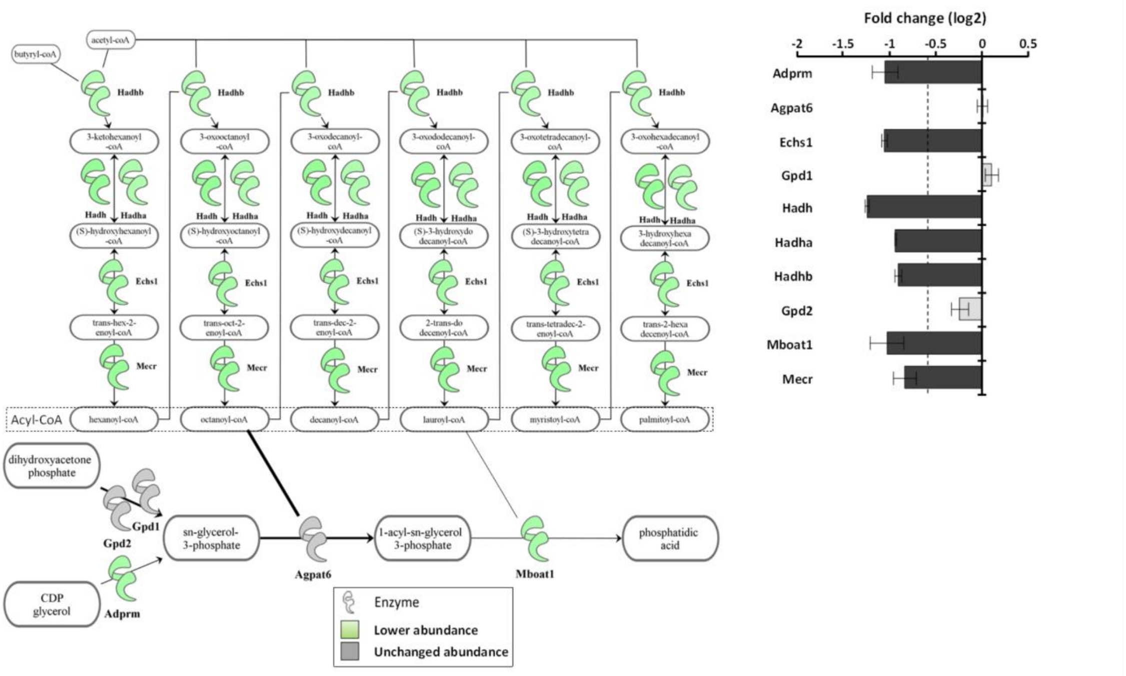

Figure S3

Figure S4. Lipid profile of *C. rodentium* (related to Figure 3B).

A. MALDI-TOF negative ion mass spectra of *C. rodentium*. The absolute abundance of the ions is shown on the y axis, and the masses of the ions are shown on the x axis. The m/z represents mass to charge ratio. **B.** Relative abundance of phosphatidylinositol detected in uninfected and infected IECs. Mann-Whitney test with p-value < 0.05. Each dot represents an individual mouse and bars the geometrical mean. Both panels are related to Figure 3B.

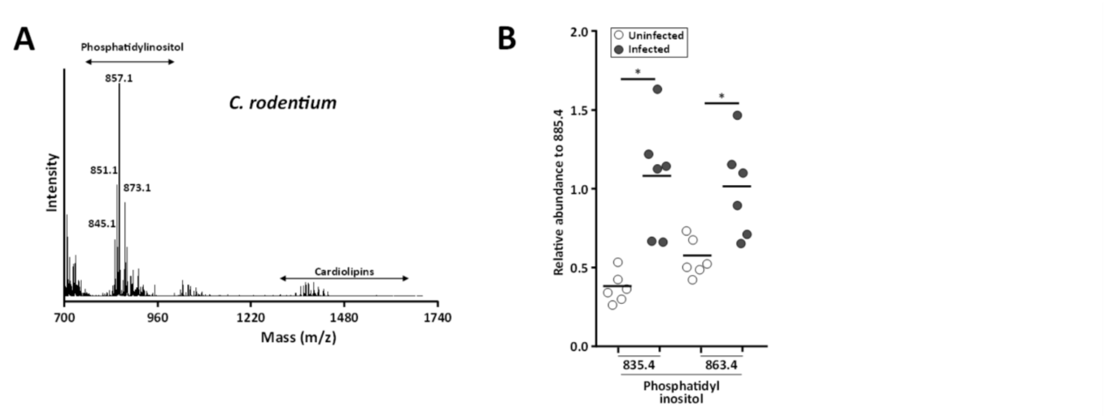

Figure S4

Figure S5. Alpha diversity of tissue-associated microbiota on day 8 p.i (related to Figure 7). Each dot represents and individual mouse and bars the mean.

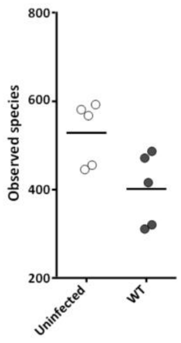

Figure S5

Table S1. List of strains (related to EXPERIMENTAL MODEL)

Strains	Characteristics	Resistance	Source
C. rodentium ICC169	Wild type *C. rodentium*	Nal^R	Schauer and Falkow, 1993
C. rodentium ICC180	*C. rodentium* ICC169::Lux	Nal^R/Kan^R	Wiles et al., 2004
C. rodentium ICC1411	*C. rodentium* ICC180*Δmap*	Nal^R/Kan^R	This study
C. rodentium ICC1412	*C. rodentium* ICC180*Δmap::map*	Nal^R/Kan^R	This study
E. coli CC118λpir	λpir lysogen of *E. coli* CC118	-	Herrero et al., 1990
E. coli CC1047	Wild type helper *E. coli* strain	-	Kaniga et al., 1991

Table S2. List of plasmid (related to Generation of *C. rodentium map* mutant)

Plasmids	Characteristics	Resistance	Source
pSEVA612S-*map*	pSEVA612S containing *map* plus 300bp upstream and downstream	Gm^R	pICC2536
pSEVA612S-*HR*	pSEVA612S containing 300bp upstream and downstream of *map,* without *map* itself	Gm^R	pICC2537
pACBSR	Plasmid containing the endonuclease I-SceI	Sm^R	Ruano-Gallego et al., 2015
pRK2013	Self-transmissible plasmid able to promote conjugal transfer of DNA	Kn^R	Figurski et al., 1979

Table S3. List of primers (related to Generation of *C. rodentium map* mutant)

Primer	Direction	Sequence 5'-3'	Enzyme	Template	Amplicon	Vector
DC074	Forward	TACTGCATGC TGT GCA AGA TCT GTG AGA AAT TGT TCA TTC AT	SphI	gDNA Map	Map + 300bp upstream and downstream	pSEVA
DC075	Reverse	TACTGAGCTC TTT ATA TTG TTA TGA TGC AAC GGT ATG CAG TC	SacI	gDNA Map	Map + 300bp upstream and downstream	pSEVA
DC084	Forward	ATAGAAAAAACATACC AAGCATTTCTCGGT	-	Conjugation colony	Map + 500bp upstream and downstream	-
DC085	Reverse	CAGGGGAGAAAATAAT AAACGAGATCC	-	Conjugation colony	Map + 500bp upstream and downstream	-
