# Supplementary material for: Citrobacter rodentium Subverts ATP Flux and Cholesterol Homeostasis in Intestinal Epithelial Cells In Vivo
Source: Cell Metab. 2017 Nov 7;26(5):738–752.e6. doi: 10.1016/j.cmet.2017.09.003 (PMC5695859; doi:10.1016/j.cmet.2017.09.003)
Supplement: Document S2. Article plus Supplemental Information [file mmc3.pdf]

Cell Metabolism

Article

Citrobacter rodentium Subverts ATP Flux and Cholesterol Homeostasis in Intestinal Epithelial Cells *In Vivo*

Graphical Abstract

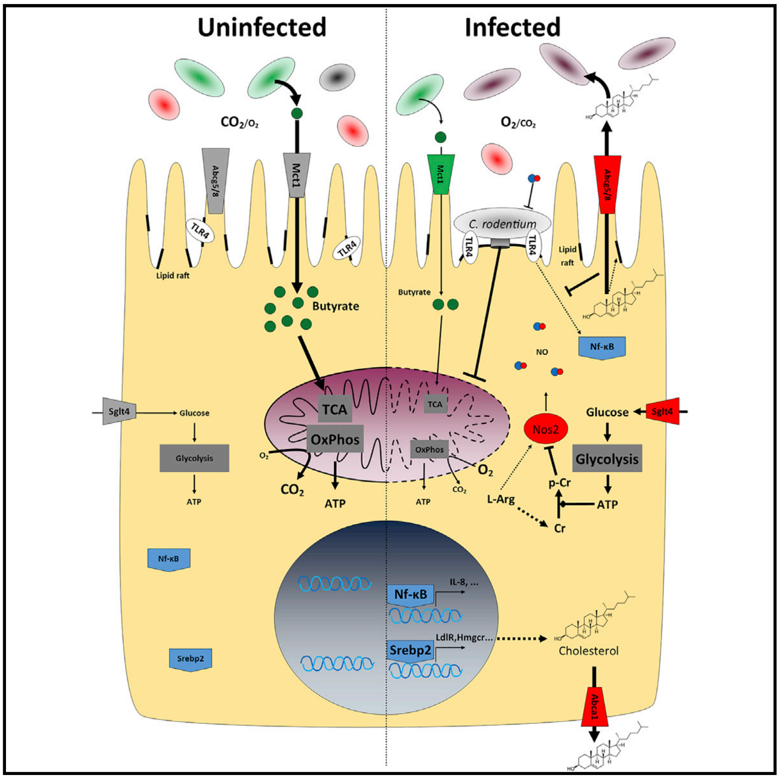

Authors

Cedric N. Berger, Valerie F. Crepin, Theodoros I. Roumeliotis, ..., Gerald J. Larrouy-Maumus, Jyoti S. Choudhary, Gad Frankel

Correspondence

jc4@sanger.ac.uk (J.S.C.),
g.frankel@imperial.ac.uk (G.F.)

In Brief

Berger et al. reveal how *C. rodentium* infection manipulates host metabolism to evade innate immune responses and establish a favorable gut ecosystem. Binding of *C. rodentium* to the gut epithelium rewires cellular bioenergetics and cholesterol metabolism, altering the composition of the gut microbiota and processes involved in fighting infection.

Highlights

- *C. rodentium* infection disrupts mitochondrial bioenergetics
- *C. rodentium* triggers aerobic glycolysis and production of phosphocreatine *in vivo*
- Srebp2 and cholesterol biogenesis and efflux are activated by *C. rodentium*
- *C. rodentium* triggers mucosal oxygenation and dysbiosis

Berger et al., 2017, Cell Metabolism *26*, 738–752
November 7, 2017 © 2017 The Author(s). Published by Elsevier Inc.
https://doi.org/10.1016/j.cmet.2017.09.003

CrossMark

CellPress

Cell Metabolism
Article

Citrobacter rodentium Subverts ATP Flux and Cholesterol Homeostasis in Intestinal Epithelial Cells *In Vivo*

Cedric N. Berger,[1,5] Valerie F. Crepin,[1,5] Theodoros I. Roumeliotis,[2,5] James C. Wright,[2,5] Danielle Carson,[1] Meirav Pevsner-Fischer,[4] R. Christopher D. Furniss,[1] Gordon Dougan,[2,3] Mally Dori-Bachash,[4] Lu Yu,[2] Abigail Clements,[1] James W. Collins,[1] Eran Elinav,[4] Gerald J. Larrouy-Maumus,[1] Jyoti S. Choudhary,[2,6,*] and Gad Frankel[1,7,*]
[1]MRC Centre for Molecular Bacteriology and Infection, Department of Life Sciences, Imperial College London, London, UK
[2]Wellcome Trust Sanger Institute, Wellcome Trust Genome Campus, Hinxton, Cambridge, UK
[3]Department of Medicine, University of Cambridge, Addenbrooke's Hospital, Cambridge, UK
[4]Department of Immunology, the Weizmann Institute of Science, Rehovot, Israel
[5]These authors contributed equally
[6]Present address: Division of Cancer Biology, The Institute of Cancer Research London, London, UK
[7]Lead Contact
*Correspondence: jc4@sanger.ac.uk (J.S.C.), g.frankel@imperial.ac.uk (G.F.)
https://doi.org/10.1016/j.cmet.2017.09.003

SUMMARY

The intestinal epithelial cells (IECs) that line the gut form a robust line of defense against ingested pathogens. We investigated the impact of infection with the enteric pathogen *Citrobacter rodentium* on mouse IEC metabolism using global proteomic and targeted metabolomics and lipidomics. The major signatures of the infection were upregulation of the sugar transporter Sglt4, aerobic glycolysis, and production of phosphocreatine, which mobilizes cytosolic energy. In contrast, biogenesis of mitochondrial cardiolipins, essential for ATP production, was inhibited, which coincided with increased levels of mucosal O_2 and a reduction in colon-associated anaerobic commensals. In addition, IECs responded to infection by activating Srebp2 and the cholesterol biosynthetic pathway. Unexpectedly, infected IECs also upregulated the cholesterol efflux proteins AbcA1, AbcG8, and ApoA1, resulting in higher levels of fecal cholesterol and a bloom of *Proteobacteria*. These results suggest that *C. rodentium* manipulates host metabolism to evade innate immune responses and establish a favorable gut ecosystem.

INTRODUCTION

The intestinal epithelium is comprised of LGR5$^+$ stem cells at the base of the crypt, proliferating transit-amplifying (TA) cells at the lower part of the crypt and a monolayer of columnar intestinal epithelial cells (IECs) that are renewed every 5–7 days (Barker, 2014). The proliferating crypt cells are believed to utilize aerobic glycolysis (the Warburg effect) by fermenting glucose to lactate (Koppenol et al., 2011).

The IECs play a key role in absorption and systemic dispersion of electrolytes, nutrients, and water from the lumen of the gut (Peterson and Artis, 2014). IECs also form a robust line of host defense against ingested pathogens, acting as a physical barrier and through detection of pathogen-associated molecular patterns via pattern recognition receptors, such as toll-like receptors (TLRs) 2 and TLR4 (Peterson and Artis, 2014). As such, the pathogen-IEC interface constitutes the battle line between the host innate immune system and the pathogen's counteracting virulence factors.

IECs have a high-energy demand and their extensive anabolic activity relies on various sources of energy. Glucose, glutamine, glutamate, and aspartate are delivered to IECs through the circulatory system (Blachier et al., 2017), while the short-chain fatty acids (SCFAs) acetate, propionate, and butyrate are absorbed directly from the gut lumen, where they are produced by the microbiota through fermentation of dietary fiber and amino acids (Neis et al., 2015). Butyrate, which in the colon is absorbed mainly via the monocarboxylate transporter 1 (Mct1), is processed via β-oxidation and feeds the tricarboxylic acid (TCA) cycle and oxidative phosphorylation in the mitochondria (Donohoe et al., 2011). Despite the growing appreciation of the role subversion of cellular metabolism plays during host-pathogen interactions (Fuchs et al., 2012), our understanding of changes to the metabolic networks of host cells during infection, particularly IECs, is incomplete.

Citrobacter rodentium is an extracellular, mouse-specific, pathogen that intimately binds the apical surface of IECs and triggers effacement of the brush border (BB) microvilli, forming attaching and effacing lesions, in a similar manner to enteropathogenic and enterohemorrhagic *Escherichia coli* (EPEC and EHEC) (Mundy et al., 2005). Moreover, by inducing extensive amplification of TA cells and inhibiting anoikis and cell detachment, *C. rodentium* induces colonic crypt hyperplasia (CCH) (Collins et al., 2014).

Following oral inoculation *C. rodentium* first resides in the cecum, before colonization spreads to the entire colonic mucosa (Wiles et al., 2004). Bacterial shedding peaks around 8 days post infection (DPI) and the infection starts to clear at around 12 DPI. Injection of bacterial effector proteins via a type III secretion system (T3SS) into IECs is the key mechanism by which of

 © 2017 The Author(s). Published by Elsevier Inc.
This is an open access article under the CC BY license (http://creativecommons.org/licenses/by/4.0/).

C. rodentium establishes infection at the epithelial surface (Mundy et al., 2005). Once in the host cytosol these effectors take control of key cell signaling processes, including actin dynamics, endosomal trafficking, and apoptosis (Wong et al., 2011).

The inflammatory nature of the infection means that *C. rodentium* interactions with IECs take place in an environment rich in cytokines (e.g., interleukin-18 [IL-18], IL-22, IL-6, IL-1β, tumor necrosis factor alpha [TNF-α], interferon-γ) and infiltrating immune cells (Collins et al., 2014). Consequently, infected IECs respond to the inflammatory signals in the gut by expressing high levels of antimicrobial peptides (Collins et al., 2014) and apical inducible nitric oxide synthase (iNOS), capable of producing nitric oxide (NO) to which *C. rodentium* is sensitive (Lopez et al., 2016; Vallance et al., 2002). Indeed, one well-characterized function of the T3SS effectors is the subversion of innate immune responses in IECs, e.g., nuclear factor κB [NF-κB] and c-Jun N-terminal kinase (Pearson et al., 2016) and the non-canonical caspase-4/11 inflammasome (Pallett et al., 2017). In addition, T3SS effectors also target mitochondrial functions. The effector Map, which acts as a guanine nucleotide exchange factor for Cdc42 (Huang et al., 2009), is targeted to the mitochondria where it induces disruption of the mitochondrial morphology and loss of mitochondrial respiratory functions (Ma et al., 2006). How the pathogen benefits from altering the function of the mitochondria, and thus the production and flow of energy in infected IECs, remains unknown.

In this study we conducted the first in-depth proteomics analysis of IECs isolated from mice infected with *C. rodentium* and reveal extensive remodeling of metabolic pathways during infection. We subsequently confirmed these findings using targeted assays. We show that *C. rodentium* infection results in significant dampening of central carbon metabolism in IECs, particularly production of mitochondrial cardiolipins. This coincided with elevated levels of O_2 above the infected IECs, confirming a previous report showing that *C. rodentium* favors oxidative metabolism *in vivo* (Lopez et al., 2016). Uniquely, we found that infected IECs upregulate cholesterol biogenesis, which was unusually accompanied by upregulation of the cholesterol efflux transporter Abca1 and AbcG8, as well as ApoA1, leading to elevated levels of fecal cholesterol. Finally, the infection-induced increase in luminal O_2 and cholesterol were reflected by the observed dysbiosis triggered by *C. rodentium* infection.

RESULTS

C. rodentium Disrupts Host Metabolic Processes and Cytoskeletal Proteins

To characterize the effect of *C. rodentium* infection on host metabolism *in vivo*, we enriched IECs from colons of *C. rodentium*-infected mice 8 DPI (five mice per group), when the pathogen is shed at ca. 10^9 per gram of stool. IECs isolated from uninfected mice were used as a control (five mice per group). Examination of IECs by microscopy and flow cytometry revealed that IEC preparations were enriched by over 90% (Crepin et al., 2015). Immunofluorescence microscopy revealed that IECs extracted from uninfected mice exhibited typical columnar shape and projection of actin-rich BB microvilli. IECs purified from mice 8 DPI were round, covered with *C. rodentium* associated with polymerized actin, and devoid of microvilli (Figure 1A).

Shotgun proteomic analysis of enriched IECs, in conjunction with isobaric labeling, allowed identification and quantification of 7,447 unique mouse proteins at false discovery rate $< 1\%$ (Table S4), 2,316 of which exhibited differential regulation in infected IECs (Log p value > 0.697, log 2 fold change (Log2FC) > 0.59) (Figure 1B). Among the top proteins with higher abundance after infection were Sglt4 (glucose transporter, Log2FC 5.11), Abca1 (intracellular cholesterol transporter, Log2FC 4.92), and iNOS (inducible nitric acid synthases, Log2FC 4.89).

KEGG pathway enrichment analysis of differentially regulated proteins revealed a striking tendency toward downregulation of pathways related to a broad range of cellular metabolic and energy homeostasis activities including the TCA cycle, oxidative phosphorylation, and lipid metabolism (Figure 1C). Consistently, 53% of the downregulated proteins were mitochondrial (Figure 1D).

C. rodentium Inhibits Feeding of the Host TCA Cycle

Previous studies have demonstrated that *C. rodentium* infection results in extensive disruption of the mitochondria (Ma et al., 2006), where ATP is produced via the TCA cycle and oxidative phosphorylation. Key mitochondrial transporters supplying substrates for the TCA cycle were in lower abundance in infected IECs, including the pyruvate transporter (Mpc1), the carnitine/acylcarnitine carrier (Cac), the 2-oxoglutarate/malate carrier (Ogcp), calcium-dependent exchanger of cytoplasmic glutamate with mitochondrial aspartate (Aralar1/2), and the citrate transporter (Sfxn5) (Figure 2A and quantification in Figure S1A).

The TCA cycle is fed by multiple metabolites including α-ketoglutarate, generated from glutamate by glutamate dehydrogenase and acetyl-CoA, generated via glycolysis and β-oxidation of butyrate and other lipids (Figure 2A). Proteins involved in β-oxidation were found in lower abundance in infected IECs (Figure S2). Moreover, the nuclear-encoded mitochondrial transcription factor Tfam, which regulates expression of the mitochondrial β-oxidation genes (Joseph et al., 2006), was found in lower abundance (Log2FC −0.8) and was predicted to be inactivated (*Z* score: −2.333, p value: 5.14×10^{-5}). In addition, the abundance of proteins involved in butyric acid (or butanoate) metabolism was also lower in infected IECs (Figure 1D), fitting with the predicted inhibition of this pathway (*Z* score: −3.656, p value: 5.32×10^{-4}).

Butyrate is one of the main substrates fueling β-oxidation and the TCA cycle in colonic IECs; importantly, the abundance of the butyrate importer Mct1/Slc16a1 and its co-factor Bsg/CD147 was lower (Log2FC −1.4 and Log2FC −1.1, respectively) in infected IECs (Figure 2B). Considering the central role butyrate plays in energizing IECs we tested experimentally whether *C. rodentium* infection inhibits butyrate uptake during *in vitro* infection of polarized Caco-2 cells using [^{14}C]sodium butyrate. This analysis revealed a 30% reduction of butyrate uptake into infected cells compared with uninfected control cells (Figure 2C).

Taken together, these data suggest that *C. rodentium* infection inhibits the supply of substrates to the TCA cycle in IECs, which is likely to impact on downstream oxidative phosphorylation and ATP production.

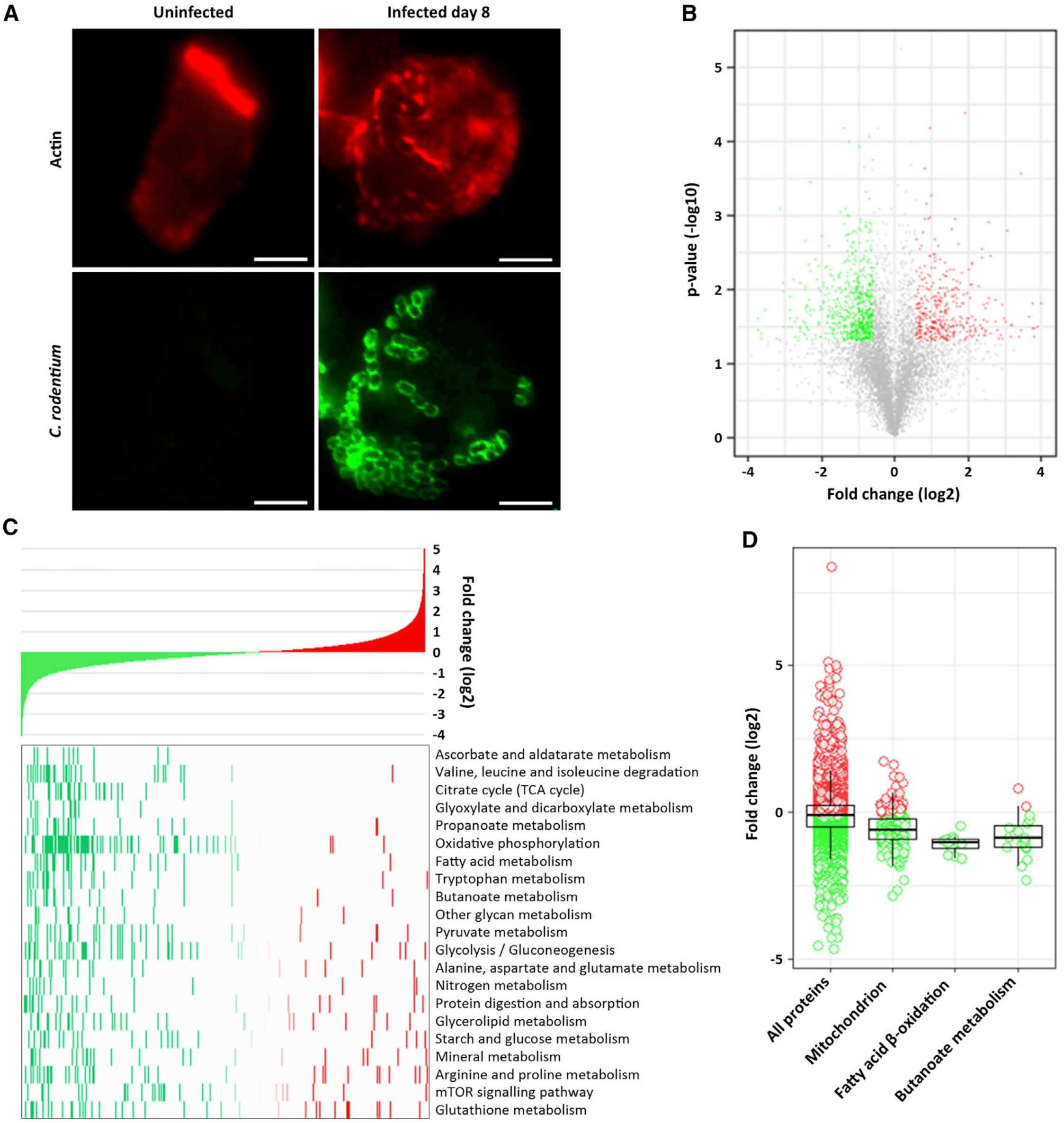

Figure 1. The Metabolic Landscape of *C. rodentium*-Infected IECs

(A) Immunofluorescence of IECs isolated from uninfected and infected mice 8 DPI and stained for *C. rodentium* (green) and actin (red). Scale bars, 5 μm.

(B) Volcano plot summarizing the differential regulation of the mouse IEC proteome during *C. rodentium* infection. Red, green, and gray dots represent proteins with higher, lower, or unchanged abundance, respectively.

(C) KEGG pathway enrichment analysis; proteins in the whole proteome are ranked according to the log2 values (top panel) from the most downregulated (green) to the most upregulated (red). Regulated proteins mapped to significantly enriched KEGG pathways are highlighted in the heatmap (bottom panel). The pathways are ranked from those that are highest statistical significant to the lowest (Benjamini-Hochberg false discovery rate [FDR] < 0.05).

(D) Boxplots illustrating the downregulation of mitochondrial proteins (MSigDB annotation), proteins involved in fatty acid, β-oxidation, and butanoate metabolism (KEGG annotation).

C. rodentium Inhibits Mitochondrial ATP Biogenesis in Infected IECs

The TCA cycle produces NADH for oxidative phosphorylation. The abundance of all the enzymes forming the TCA cycle and most of the proteins in the electron transfer chain was lower in *C. rodentium*-infected IECs compared with control IECs (Figure 2D and quantification in Figure S1B). Oxidative phosphorylation is dependent on the inner mitochondrial membrane lipid cardiolipin (ca. 20% of mitochondrial lipid content), which is essential for generating the electrochemical gradient used for ATP production (Paradies et al., 2014). Cardiolipins are synthesized in the mitochondrial inner membrane by conversion and modification of phosphatidic acid (PA), which is transferred from the mitochondrial outer membrane by a complex comprising Ups1/Preli and Mdm35/Triap (Miliara et al., 2015; Yu et al., 2015).

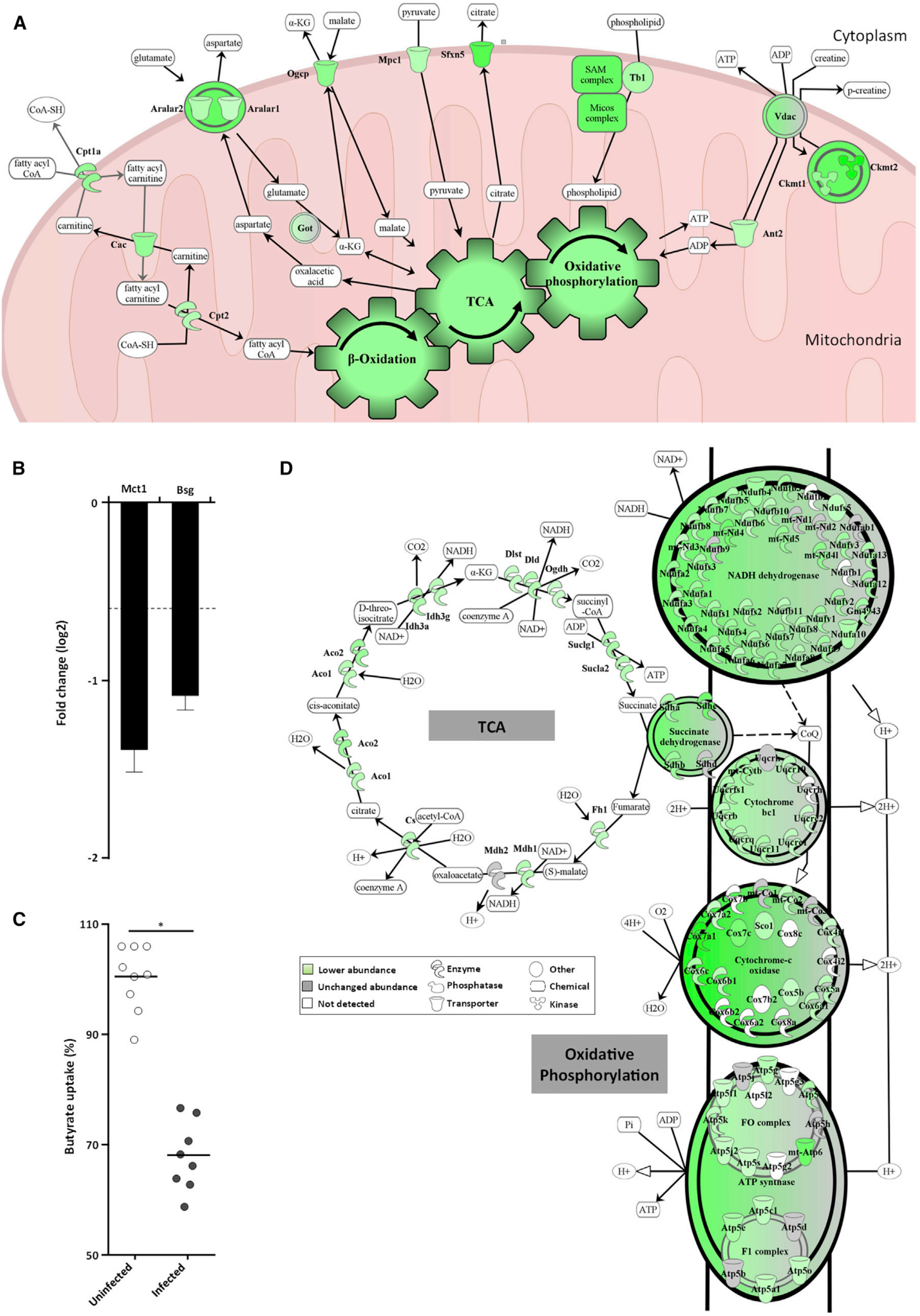

(legend on next page)

We found all five enzymes generating long-chain acyl-CoA and PA in lower abundance in infected IECs (Figure S3), which may result in accumulation of low-molecular-weight phospholipids, including phosphatidylinositols (PIs), and cardiolipins. Moreover, while Crls1, which generates immature cardiolipin, was in higher abundance (Figure 3A and quantification in Figure S1C), the final maturation steps are likely to be impaired due to lower abundance of the mitochondrial and cytosolic cardiolipin maturation enzymes, Mlclat1 and Alcat1, respectively, and the unchanged abundance of the phospholipase iPla2, which digests monolysocardiolipin into dilysocardiolipin (Figure 3A and quantification in Figure S1C). Moreover, the abundance of Mdm35/Triap was lower in infected IECs (Log2FC −0.67). Based on these observations we hypothesized that infected cells will either accumulate low-molecular-weight cardiolipins or decrease the pool of cardiolipins.

By applying a lipidomics fingerprint technique to infected IECs we were able to detect cardiolipins and PIs and to quantify their abundance (Figure 3B). As a control, total lipid extracts from *C. rodentium* were analyzed to confirm the specificity for eukaryotic PIs and cardiolipins (Figure S4A). As predicted from protein abundance measurements, low-molecular-weight PIs, present at *m/z* 835.4 and 863.4 (Figure 3B), was in higher abundance in infected IECs (Figure S4B). In addition, cardiolipins of the highest molecular weights, present at *m/z* 1,570.1 and 1,598.1, were in lower quantities in infected IECs, while cardiolipins of lower molecular weights, present at *m/z* 1,396.0 and 1,424.0, were in higher abundance (Figure 3C), suggesting that accumulation of immature mitochondrial cardiolipins disturbs oxidative phosphorylation during *C. rodentium*.

The T3SS effector Map is responsible, at least in part, for mitochondrial disruption in the colonic IECs (Ma et al., 2006). We therefore reasoned that as oxygen consumption by oxidative phosphorylation in IECs would be more efficient following infection with a *map* mutant (partial disruption of the mitochondria) compared with infection with wild-type (WT) *C. rodentium* (extensive disruption of the mitochondria), the apical surface of infected IECs would be more hypoxic following infection with the former. To test this experimentally, we infected mice with bioluminescent WT *C. rodentium* or a *C. rodentium map* mutant as reporters for surface oxygen concentration (as luciferase activity is dependent on the supply of O_2 to the epithelium [Ghisla et al., 1978]). At 6 DPI both strains were shed at equal numbers (Figure 4A). Moreover, the number of tissue-associated *C. rodentium* (Figure 4B), the magnitude of CCH (Figure 4C), and the level of Ki-67 straining, a marker of proliferating cells (Figure 4D), were similar at 8 DPI with either the WT or the *map* mutant strains. However, the bioluminescence signal was significantly lower following infection with *C. rodentium* Δ*map* compared with infection with WT *C. rodentium* (Figure 4E). Upon complementation of the *map* mutant the increased bioluminescent signal was restored (Figure 4E). These results show that infection with WT *C. rodentium* results in oxygenation of the apical surface of infected IECs independently of CCH.

C. rodentium Infection Triggers Biogenesis of Phosphocreatine

While the proteomic and lipidomics analyses showed that the mitochondria are dysfunctional during *C. rodentium* infection, cytosolic glycolysis seems to be functioning, as key enzymes from throughout the glycolysis pathway are either unchanged or exhibit increased abundance during infection (Figure 5A and quantification in Figure S1D). Moreover, the infected IECs adapted to the lack of mitochondrial ATP production by specifically upregulating the basolateral sugar importer Sglt4 (the abundance of the glucose transporter Glut1 did not change during infection) (Figure 5A and quantification in Figure S1D).

Next, we analyzed how the glycolysis-generated ATP is efficiently distributed to subcellular sites of energy utilization. One such go-between is phosphocreatine (PCr), which is generated by phosphorylation of creatine (Cr) by creatine kinases (CKs) (Wallimann et al., 2011). While biogenesis of Cr (as well as ornithine/spermidine) is mediated via degradation of L-arginine by Gatm, iNOS uses L-arginine as a substrate for generation of NO. Importantly, the abundance of both Gatm and iNOS was significantly higher in infected IECs (Figure 5B and quantification in Figure S1E). As NO is highly bactericidal, it is possible that *C. rodentium* disrupts the mitochondria as a means to shift cellular utilization of L-arginine toward generation of Cr and away from production of NO. This hypothesis is supported by the fact that, based on the fold change of its target proteins, the transcription factor Nrf2, which is activated by NO (Kvandova et al., 2016), was predicted to be strongly inhibited (Z score: −4.74, p value: -7.22×10^{-10}) in infected IECs. Moreover, *C. rodentium* intimately colonize IECs expressing high levels of apical iNOS (Vallance et al., 2002).

Conversion of Cr to PCr can occur in the mitochondria, by the mitochondrial CKs Cktm1 and Cktm2, or in the cytosol via CK-m and CK-b, which are directly associated with the glycolytic enzymes producing ATP (Joseph et al., 1997). The abundance of CK-m and CK-b was unchanged during infection, while the abundance of Cktm1 and Cktm2 was lower (Figure 5B and quantification in Figure S1E). Consistently, the inner membrane mitochondrial ATP exporter (Ant), which is tightly coupled to Cr phosphorylation, and the mitochondrial outer membrane voltage-dependent anion channel (Vdac), which exports PCr into the cytosol, were in lower abundance following infection (Figure 2A and quantification in Figure S1A). Taken together, this suggests that, in *C. rodentium*-infected IECs, L-arginine is mainly catabolized to Cr, which is converted by cytosolic CKs to PCr. We confirmed this experimentally using LC-MS-based metabolomic analysis, which revealed higher levels of the Cr

Figure 2. The Effect of *C. rodentium* Infection on Mitochondrial Functions

(A) Schematic representation of mitochondrial transporters affected by *C. rodentium* (quantification is shown in Figure S1A).

(B) Bar plot showing the relative abundances of the butyrate transporter Mct1 and its co-factor Bsg during infection. Data are represented as mean ± SD.

(C) [^{14}C]Sodium butyrate uptake into uninfected Caco-2/TC7 cells or cells infected for 2.5 hr with *C. rodentium*. *Mann-Whitney test with p value < 0.05. Each dot represents an individual well and bars show the means.

(D) Schematic representation of the mitochondrial TCA cycle and respiratory chain with the affected proteins during infection (quantification is shown in Figure S1B).

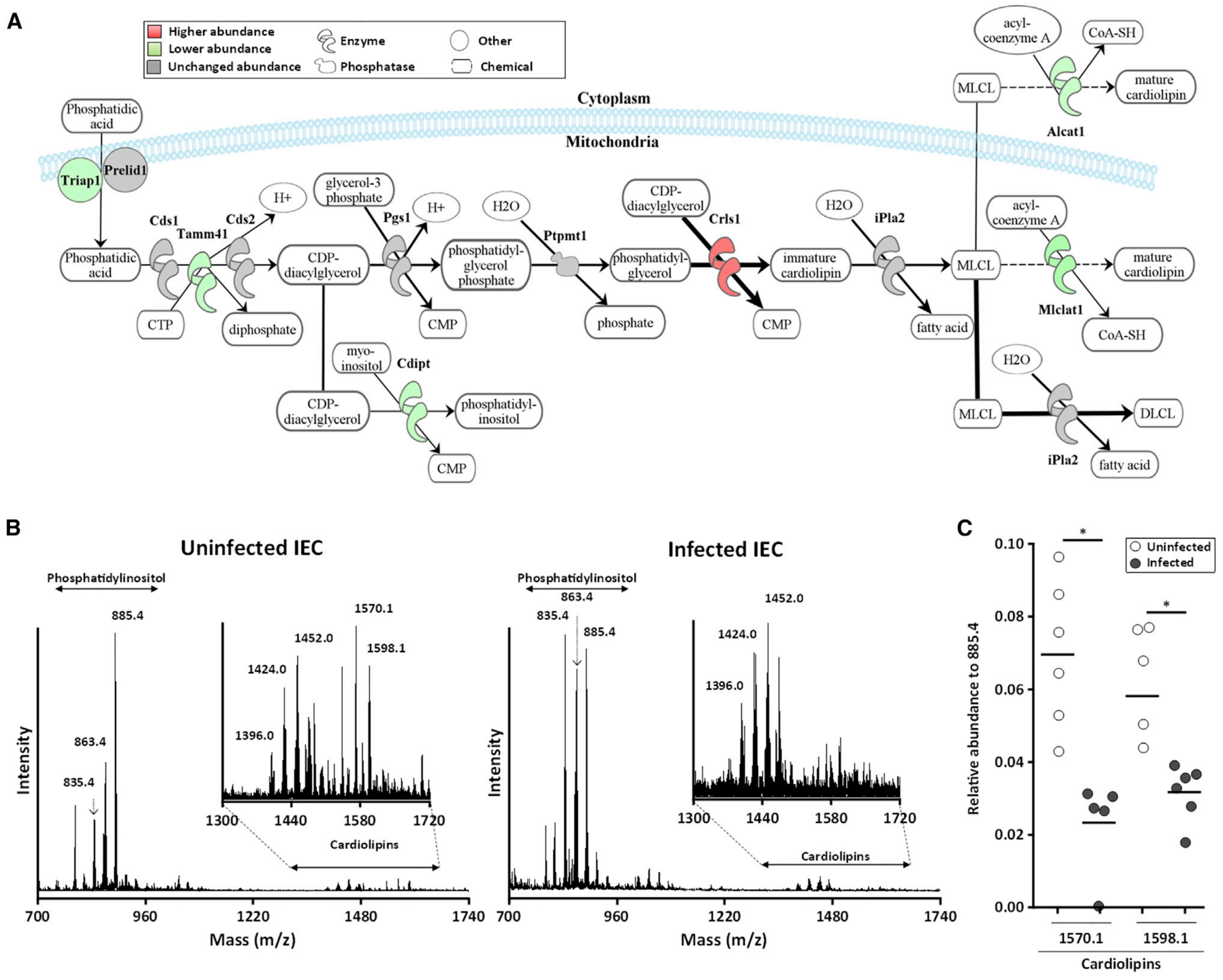

Figure 3. Cardiolipin Biogenesis in IECs during *C. rodentium* Infection

(A) Schematic representation of the regulated proteins involved in cardiolipin biogenesis (quantification is shown in Figure S1C). Proteins below the significant value (log2 fold change >0.59 or <−0.59) are shown in gray. MLCL, monolysocardiolipin; DLCL, dilysocardiolipin.

(B) MALDI-TOF mass spectra of uninfected (left panel) and infected IECs (right panel) showing the negative ion mass spectra using the DHB matrix solubilized at 10 mg/mL (mass spectra of *C. rodentium* are shown in Figure S4A). The absolute abundance of the ions is shown on the y axis, and the masses of the ions are shown on the x axis. The *m/z* represents mass to charge ratio.

(C) Relative abundance of cardiolipins detected in uninfected and infected IECs (relative abundance of phosphatidyl inositol is shown in Figure S4B). Mann-Whitney test with *p < 0.05. Each dot represents an individual mouse and bars geometric means.

precursor guanidinoacetate (6.42 FC), Cr (2.16 FC), PCr (2.93 FC) and the breakdown produce of PCr, creatinine (2.37 FC; Figure 5C), as well as spermidine (91.77 FC; Figure 5D), in infected IECs.

C. rodentium Triggers Simultaneous Cholesterol Biogenesis and Cholesterol Efflux

While a fall in the PCr:Cr ratio activates the AMP-activated protein kinase (Ampk), a crucial cellular energy sensor (Hardie et al., 2012), IECs infected with *C. rodentium* exhibit an increased PCr:Cr ratio, suggesting that Ampk is not activated. Indeed, while, the abundance of Ampk-α was similar in uninfected and infected IECs, the abundance of Ampk-β and Υ subunits was lower in infected cells (Figure 6A and quantification in Figure S1F). Moreover, the abundance of Lkb1, which is the main kinase that phosphorylates and activates Ampk-α (Hardie, 2014), was lower in infected IECs (Figure 6A and quantification in Figure S1F). Consistently, western blotting using anti-phospho Ampk-α antibodies revealed lower levels of Ampk-α phosphorylation in infected cells (Figure 6B). Moreover, based on the fold change of its target proteins, the transcription factor p53, which, once activated by Ampk, inhibits cell proliferation (Jones et al., 2005), is predicted to be inhibited (*Z* score: −4.825, p value: 2.19×10^{-29}). In addition, we observed increased abundance of Acaca/b, which catalyzes the carboxylation of acetyl-CoA to malonyl-CoA, and decreased abundance of Mlycd, which catalyzes the conversion of malonyl-CoA back to acetyl-CoA (Figure 6A and quantification in Figure S1F), which goes against

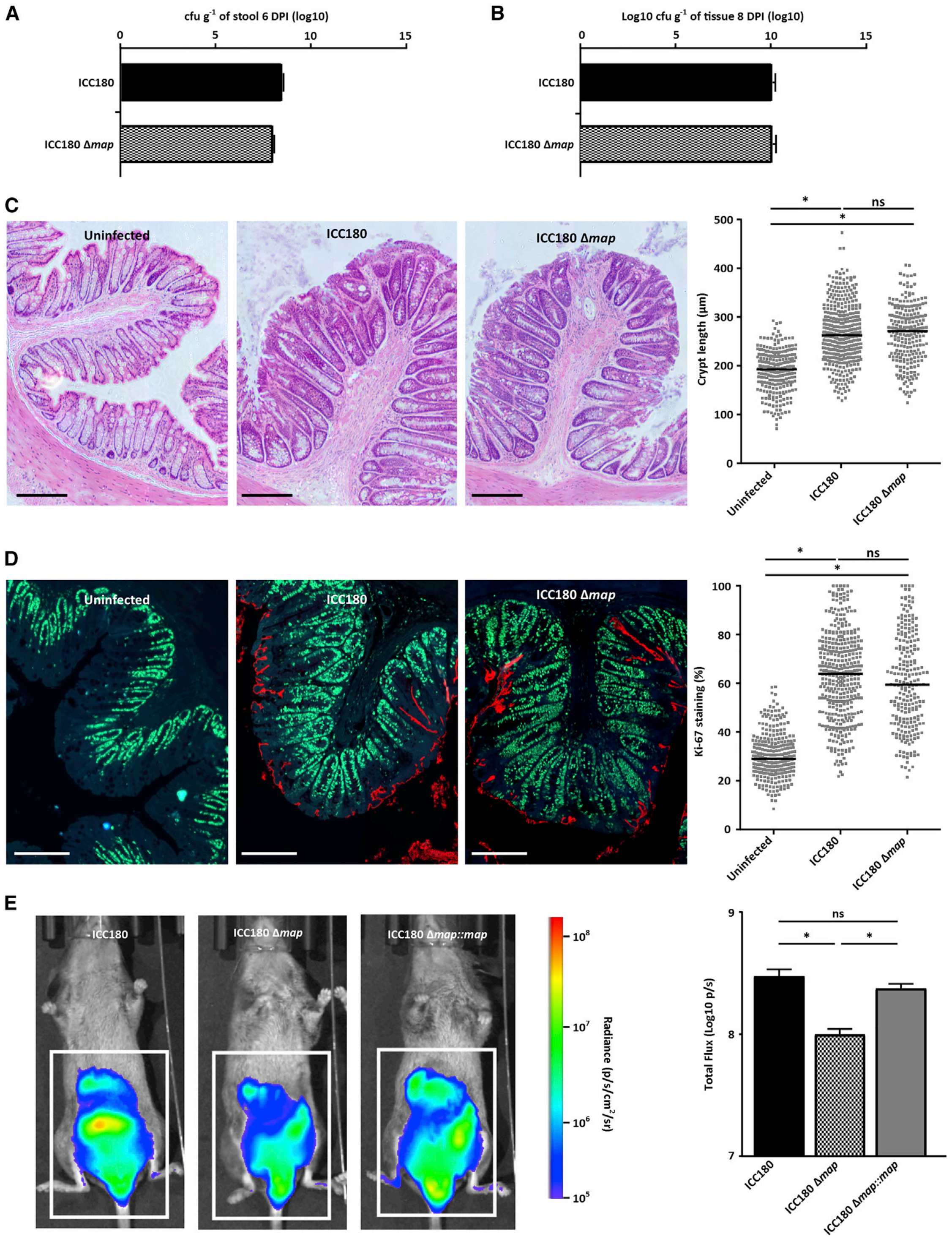

Figure 4. Infection with *C. rodentium* Triggers Mucosal Oxygenation

(A) *C. rodentium* shedding from mice infected with either bioluminescent WT (ICC180) or the isogenic Δ*map* mutant. Data are represented as mean ± SD.

(B) Level of mucosal-associated WT *C. rodentium* or *C. rodentium* Δ*map*. Data are represented as mean ± SD.

(C) WT *C. rodentium* and the Δ*map* strains trigger similar levels of CCH. Crypt measurements were taken from H&E-stained colonic sections (representative images are shown). Scale bars, 200 μm. The graph shows measurement of individual crypt lengths. Bars represent means; *p ≤ 0.0001.

(legend continued on next page)

the activation of Ampk (Wolfgang and Lane, 2006). Significantly, while Ampk inhibits cholesterol synthesis, the proteomic analysis suggested that cholesterol biosynthesis was upregulated (Figure 6B and quantification in Figure S1G), with most of the enzymes driving cholesterol biogenesis found in higher abundance, although DHCR7, which catalyzes the conversion of 7-dehydrocholesterol to cholesterol, was in lower abundance (Figure 6B and quantification in Figure S1G). In agreement with upregulation of cholesterol biosynthesis, the abundance of the Ldl receptor (Ldl-R, Log2FC 1.31) and Pcsk9 (Log2FC 1.97), involved in cholesterol uptake and receptor recycling, were elevated in infected IECs. These responses are typical of cells suffering sterol deficiency (Spann and Glass, 2013). This hypothesis was supported by the fact that, based on the fold change of its target proteins, the transcription factor Srebp2, which upregulate expression of genes involved in cholesterol biosynthesis and uptake (Spann and Glass, 2013), was predicted to be strongly activated (*Z* score: 2.032, p value: 2.28×10^{-8}) in infected IECs.

Under resting conditions Srebp2 localizes to the ER, however, sterol depletion promotes ER-to-Golgi transport of the sterol regulator Scap together with Srebp2, where Srebp2 undergoes proteolytic cleavage leading to nuclear translocation of its soluble N terminus and subsequent expression of sterol-regulated genes such as *Hmgcr* and *ldlR* (Spann and Glass, 2013). Western blotting of IECs purified from infected and uninfected mice confirmed that Srebp2 was specifically cleaved and activated during *C. rodentium* infection (Figure 6D).

Under Srebp2 activation conditions, processes involved in cholesterol efflux are inhibited (Spann and Glass, 2013). Unexpectedly, in infected IECs we found significantly higher abundance of the major basolateral cholesterol efflux transporter Abca1 (Log2FC 4.92), as was the abundance of the cholesterol binding protein Apoa1 (Log2FC 0.89); the apical cholesterol heterodimeric transporter Abcg5 and Abcg8 was not found in the proteome. We used western blotting to validate the induction of AbcA1 expression and to test whether Abcg8 is expressed in infected IECs. While Abca1 and Abcg8 were barely detectable in control IECs, they were in higher abundance in infected cells (Figure 6E). Cholesterol secreted via Abca1/Apoa1 is excreted in feces via the reverse cholesterol transport (via the liver) while trans-intestinal cholesterol excretion is mediated by Abcg5/8 (Hong and Tontonoz, 2014). We therefore investigated the consequences of the apparent increase in cholesterol efflux by measuring its levels in feces (Figure 6F). This revealed a 67% increase in fecal cholesterol 8 DPI ($p < 0.0001$).

We hypothesized that the combined elevated levels of fecal cholesterol and mucosal oxygen would impact on the composition of the colonic mucosal-associated microbiota. Phylogenetic analyses revealed no significant changes in alpha diversity (Figure S5), while principal-components analysis of operational taxonomic units at the genus level showed a 70.3% separation between infected and uninfected microbiomes (Figure 7A). In particular, the abundance of butyrate-producing commensals (*Roseburia*, *Coprococcus* and *Odoribacter* genera and members of the Lachnospiraceae family) as well as *Firmicutes*/*Bacilli* and, to a lesser extent, *Firmicutes*/*Clostridia* and *Firmicutes*/*Erysipelotrichi*, was significantly reduced in infected mice (Figures 7B and 7D), which is consistent with the oxygenation of the apical IEC surfaces. A decline in *Bacteroidetes* and *Tenericutes* was also observed (Figures 7B and 7E). In contrast, the facultative aerobes *Proteobacteria* became the dominant phylum among mucosal-associated bacteria during infection (Figures 7B and 7C), largely due to IEC-associated *C. rodentium* (Figure 7C). Interestingly, genera not detected in uninfected mice expanded significantly during infection (e.g., *Dickeya*, *Cronobacter*, *Erwinia*, *Klebsiella*, *Pantoea*, *Serratia*, and *Trabulsiella*) (Figure 7C), suggesting that, by increasing the availability of O_2 and/or cholesterol, *C. rodentium* infection provides a beneficial niche for these commensal bacteria. Notably, *Serratia*, *Dickeya*, and *Erwinia* have previously been described to thrive on and metabolize cholesterol (Caspi et al., 2016; Garcia et al., 2012).

Taken together, the apparent "confused" cellular behavior in relation to cholesterol homeostasis suggests that *C. rodentiuum* and the host clash over the control of cholesterol biogenesis and efflux, which impacts on the composition of the microbiota.

DISCUSSION

Enteric bacterial pathogens and the infected host battle for control of the gut ecosystem. This battle is classically thought to occur between the host's innate and acquired immune systems and counteracting bacterial virulence factors. However, the influence of infection on host cell metabolism is an underappreciated aspect of host-pathogen interactions. In this study we employed an unbiased quantitative shotgun proteomic screen, targeted metabolomics, and lipidomics to define the metabolic responses of mouse colonic IECs to *C. rodentium* infection, which provides a powerful and physiologically relevant infection model (Collins et al., 2014).

While the fact that *C. rodentium* infection triggers substantive disruption of the mitochondria is well established (Ma et al., 2006), the advantage this offers to the pathogen is unknown. Study of bacterial interference with the mitochondria has previously focused on apoptosis (Giogha et al., 2014). In this study we show that *C. rodentium* infection causes shut down of mitochondrial ATP production, a switch to aerobic glycolysis and significant reduction in the levels of host high-molecular-weight cardiolipins, lipids essential for efficient oxidative phosphorylation and maintenance of mitochondrial integrity (Ren et al., 2014). In addition, using bioluminescent reporter strains, we show that infection with WT *C. rodentium* leads to increased oxygenation of the mucosal surface, likely due to disruption of mitochondrial respiration. This observation is consistent with previous work, which suggests that *C. rodentium* performs

(D) Similar levels of Ki-67 straining were observed following infection with the WT and the Δ*map* strains. Scale bars, 200 μm. The graph shows the ratio of Ki-67-positive cells over total crypt length. The graph shows measurement of Ki-67 staining in individual crypt. Bars represent means; $^*p \leq 0.0001$.

(E) Bioluminescence levels are lower in mice infected with the Δ*map* compared with those infected with the WT or complemented strains. The color scale bar indicates relative signal intensity (as photons s^{-1} cm^{-2} sr^{-1}). The graph shows quantification of total flux (p/s) output from a defined area (white rectangular outline; 3.5×5 cm) of at least three mice per group. *t test with p value < 0.05; n.s., not significant. Data are represented as mean ± SEM.

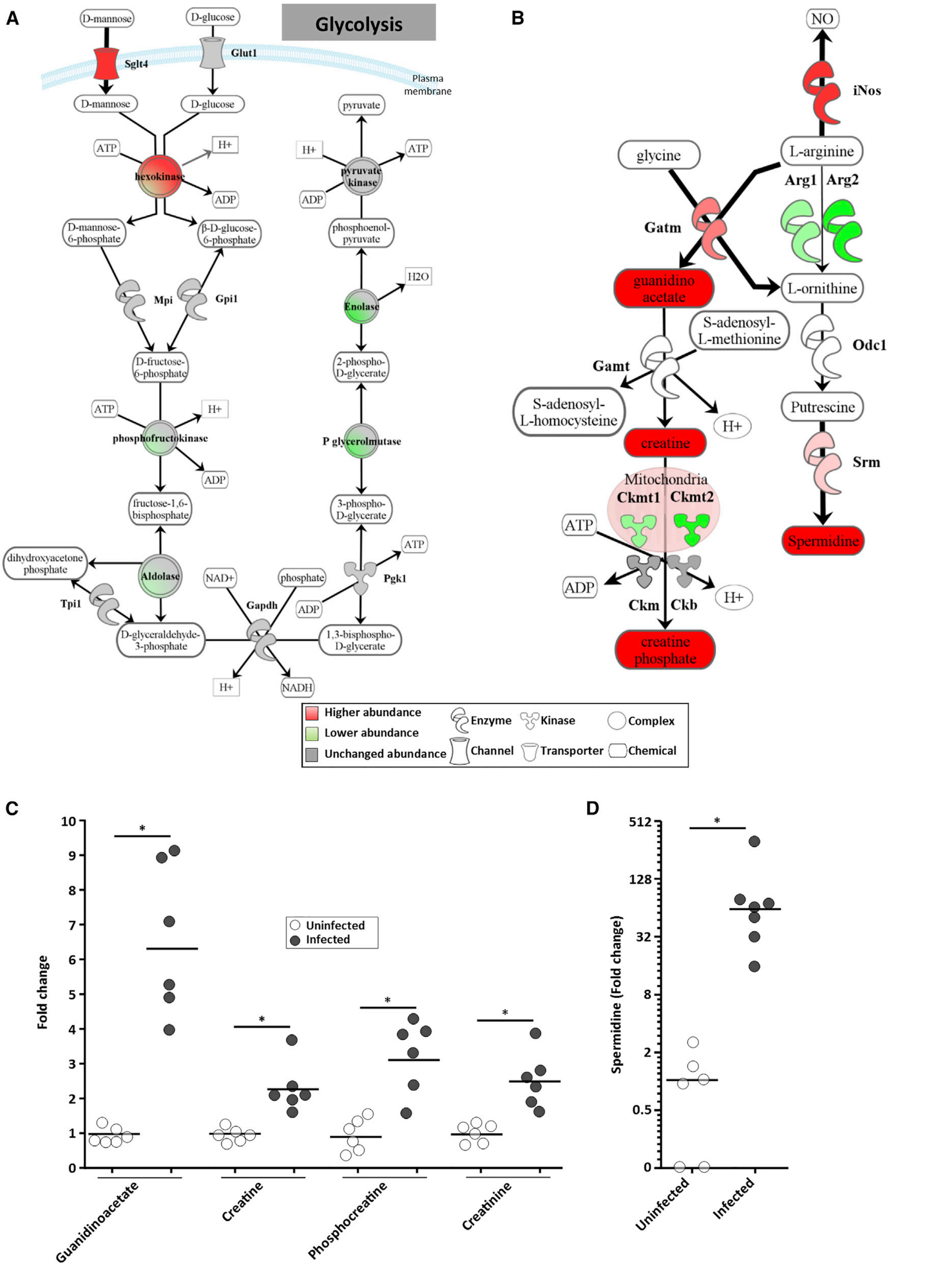

Figure 5. *C. rodentium* Triggers Production of Phosphocreatine

(A) Schematic representation of the regulated proteins in the sugar import and glycolysis pathway. *C. rodentium* induces increased abundance of the sugar transporter Sglt4, feeding glycolysis, which remained functional during infection (quantification is shown in Figure S1D).

(legend continued on next page)

oxidative metabolism *in vivo* (Lopez et al., 2016). However, while Lopez et al. suggested that *C. rodentium* triggers CCH as a means to extract oxygen, we find that oxygenation of the mucosal surface occurs independently of CCH. The disparity between these results is likely due to the use of a triple *map/espH/cesF C. rodentium* mutant by Lopez et al., which colonizes the gut inefficiently. Therefore, questions remain as to the relationship between CCH and oxygenation of the mucosal surface during *C. rodentium* infection. Of note, oxygen availability has been shown to be a key environmental cue for expression or activation of the T3SS in EHEC (Carlson-Banning and Sperandio, 2016) and *Shigella flexneri* (Marteyn et al., 2010).

The proteomics analysis reveals that the abundance of many plasma membrane and mitochondrial lipid and carbohydrate transporters, which feed the TCA cycle, are significantly reduced in infected IECs. This results in reduced butyrate uptake by IECs infected with *C. rodentium*, similar to previously reported data during EPEC infection (Borthakur et al., 2006). This observation is consistent with the lower abundance of the butyrate importer Mct1 and its co-factor Bsg/CD147. Moreover, the microbiome analysis reveals reduction in the abundance of butyrate-producing commensals, which may further impact on the ability of infected IECs to derive energy from luminal SCFAs. The reduction in butyrate-producing commensals may be due to multiple factors, including *C. rodentium*-induced generation of antimicrobial peptides (Collins et al., 2014) and *C. rodentium*-induced oxygenation of the colonic mucosa, which could impact on the viability of anaerobic members of the microbiota. Of note, Lopez et al. (2016) found increased abundance of anaerobic commensals (e.g., *Clostridia*) within the oxygenated mouse gut. The differences between the two studies are likely due to the fact that while we quantified the abundance of mucosal-associated commensals, Lopez et al. extracted DNA for microbiome analysis form the colon content.

Importantly, while the supply of mitochondrial derived ATP seemed to be inhibited, infected IECs do not present signs of ATP starvation (no signs of Ampk activation). Instead, IECs adapt to *C. rodentium* infection by increasing the abundance of sugar transporters that could feed aerobic glycolysis. The transition from oxidative phosphorylation to glycolysis is reminiscent of cancer cells and of classically activated macrophages (M1), which rely on aerobic glycolysis for energy, a phenomenon known as the "Warburg effect" (Koppenol et al., 2011). Notably, the fact that inhibition of glycolysis in transformed cells can re-activate oxidative phosphorylation suggests that the mitochondria in these cells are not damaged (Fantin et al., 2006). In contrast, *C. rodentium* disrupts the structure of IEC mitochondria, likely locking infected cells in aerobic glycolysis and forcing them to produce creatine.

Coupled with the aerobic glycolytic program, the abundance of enzymes involved in L-arginine degradation, which leads to biosynthesis of Cr/PCr and spermidine was higher in infected IECs. We confirmed experimentally the presence of elevated levels of these metabolites. Although we detected a ca. 100-fold increase in the level of spermidine in infected IECs, we are unable to conclude that this is due solely to increase spermidine production by infected IECs, as spermidine can also be generated by *C. rodentium* and the commensal flora. Importantly, in addition to being a substrate for Cr biogenesis, L-arginine is also used by iNOS, the protein with the sixth highest FC in response to infection. The inflammatory responses to *C. rodentium* infection leads to robust decoration of the apical surface of IECs with iNOS (Vallance et al., 2002); yet, although sensitive to NO (Vallance et al., 2002), *C. rodentium* thrives while forming intimate attachments with the plasma membrane of IECs. We therefore suggest that by triggering disruption of the mitochondria *C. rodentium* forces IECs to tilt the balance away from iNOS and NO production toward Gatm and Cr and polyamines, which themselves inhibit iNOS (Southan et al., 1994). This rebalancing may represent an immune evasion strategy. Importantly, at 14 DPI the abundance of Gatm returned to the pre-infection level, while the abundance of iNOS remained at the level seen 8 DPI, which could potentially contribute to *C. rodentium* clearance. Indeed, iNOS-deficient mice display a small but significant delay in bacterial clearance (Vallance et al., 2002). To the best our knowledge, our study is first to show that such an evasion strategy might occur *in vivo* in IECs. Previous studies have demonstrated that, while infecting the macrophage cell line RAW264.7 *in vitro*, *Salmonella typhimurium* upregulates expression of Arg2 to divert arginine away from iNOS (Lahiri et al., 2008). While we detected lower abundance of both Arg1 and Arg2 in IECs during *C. rodentium* infection, subversion of substrates from iNOS might be a common mechanism of innate immune evasion by pathogenic bacteria.

While lipid biogenesis in general was downregulated in infected IECs, one of the most conspicuous consequences of *C. rodentium* infection was activation of Srebp2 and cholesterol biogenesis, despite the high-energy cost involved. Moreover, although seeming to be in limited supply, the available acetyl-CoA appeared to be diverted to cholesterol biogenesis. This is the first time the cholesterol biosynthetic pathway has been shown to be induced in IECs response to an enteric infection.

Our current understanding of the function cholesterol plays in innate immunity mainly comes from studies of macrophages, where a positive feedback loop augments inflammatory responses. Macrophages containing elevated levels of cholesterol, e.g., in *abca1* knockdown cells or in hypercholesterolemia not only contain higher levels of TLR4 and TLR9, but are also hyper-responsive to lipopolysaccharide as well as to TLR2, TLR7, and TLR9 agonists (Tall and Yvan-Charvet, 2015). As TLR signaling, e.g., IL-6 or TNF-α, triggers activation of Srebp2 (Gierens et al., 2000) and decreases cholesterol efflux, the cholesterol content of lipid rafts increases, which further amplifies

(B) Schematic representation of the regulated proteins in the phosphocreatine pathway. L-Arginine is diverted toward production of spermidine, creatine, and phosphocreatine (quantification is shown in Figure S1E).

(C) Relative abundance of creatine and creatine derivatives detected in uninfected and infected IECs. Mann-Whitney test with p value < 0.05. Each dot represents an individual mouse and bars show the geometric means. *Mann-Whitney test with p value < 0.05.

(D) Relative abundance of spermidine detected in uninfected and infected IECs. Each dot represents an individual mouse and bars show the geometric means. *Mann-Whitney test with p value < 0.05.

CellPress

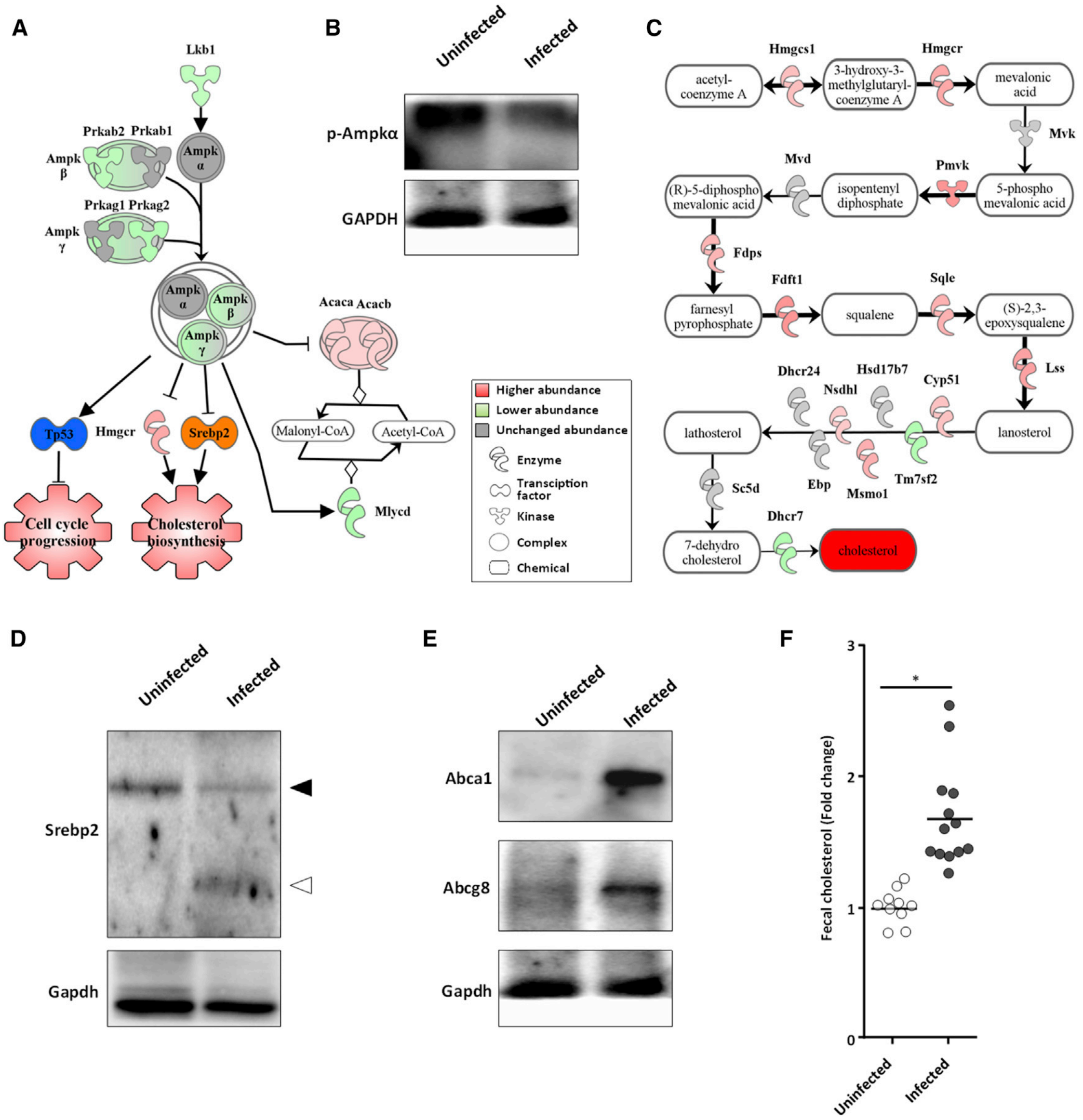

Figure 6. *C. rodentium* Triggers Production and Secretion of Cholesterol

(A) Schematic representation of the Ampk-regulated proteins and downstream pathways, suggesting that Ampk is inactive. The transcription factor TP53 was predicted to be inhibited (blue), whereas Srebp2 was predicted activated (orange), promoting cell-cycle and cholesterol biosynthesis, respectively (quantification is shown in Figure S1F).

(B) Phosphorylation of Ampkα in control and infected IECs.

(C) Schematic representation of the regulated proteins in the cholesterol biosynthetic pathway showing a global increased of enzyme abundance (quantification is shown in Figure S1G). Proteins below the significant value (log2 fold change >0.59 or <−0.59) are shown in gray.

(D) Western blot of Srebp2 and Gapdh on uninfected and infected IECs showing that Srebp2 (full arrowhead) is cleaved and activated in infected cells (open arrowhead). A pool of three mice was used for the western blot.

(E) Western blots of Abca1 and Abcg8 in uninfected and *C. rodentium*-infected IECs.

(F) Level of fecal cholesterol measured in uninfected and infected mice. *Mann-Whitney test with p value < 0.05. Each dot represents an individual mouse and bars show the geometric means.

activation of TLRs and NF-κB signaling (Tall and Yvan-Charvet, 2015). Although no equivalent data are available for IECs, activation of the cholesterol biosynthetic pathway may represent an important arm of the innate immune response to bacterial infection at mucosal surfaces. Indeed, the TLR adaptor, MyD88, is essential for host survival and optimal immunity following *C. rodentium* infection (Collins et al., 2014). Moreover, *C. rodentium* infection triggers rapid NF-κB nuclear translocation and robust recruitment of macrophages and neutrophils, which is diminished in TLR4-deficient mice. In addition, TLR2−/− mice succumb to *C. rodentium* infection (Collins et al., 2014). Consistent with this, a large proportion of the *C. rodentium*

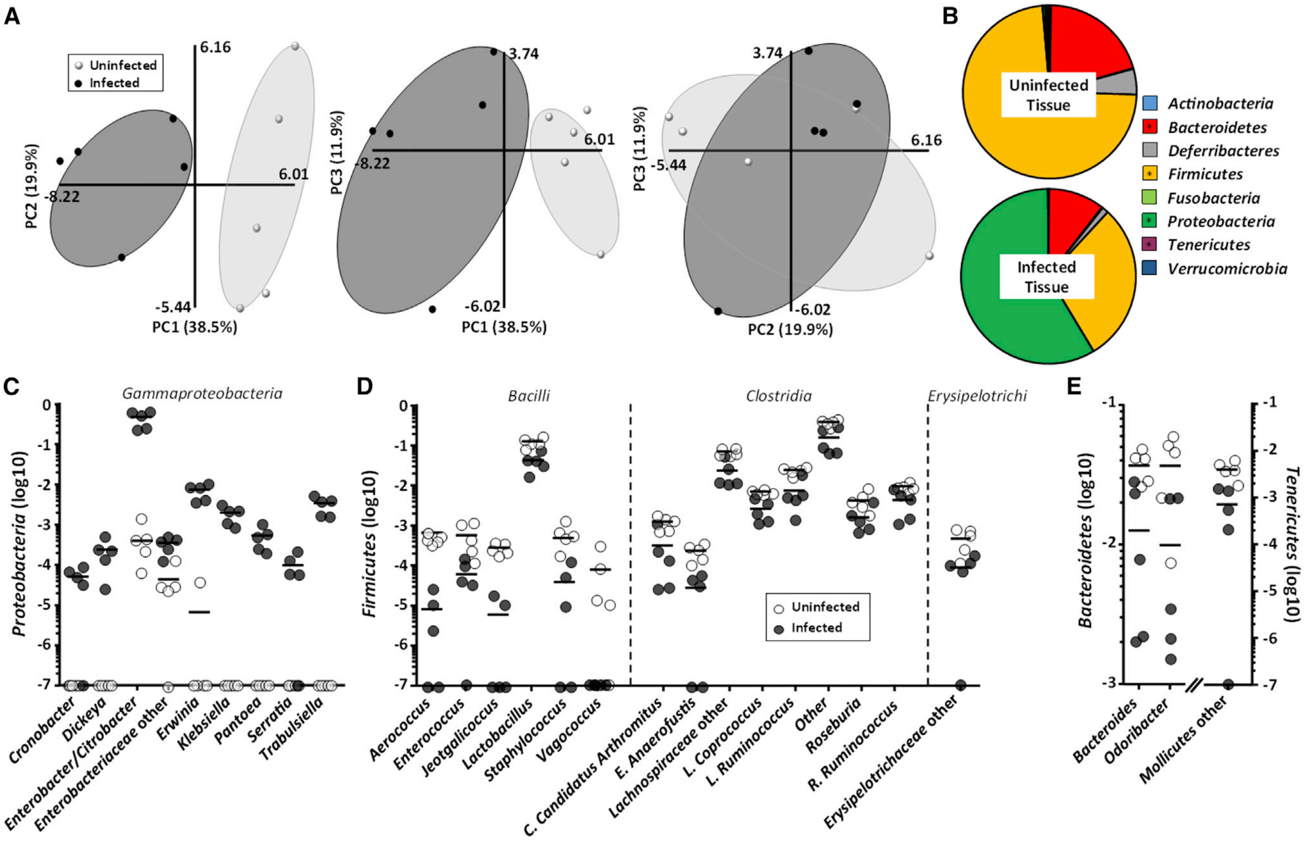

Figure 7. Impact of *C. rodentium* on the Tissue-Associated Microbiota

(A) Three-dimensional principal-components analysis (PCA) of tissue-associated microbiota at the genus level (observed species are shown in Figure S5).

(B) Relative abundance (average) of the different phyla found in tissue-associated microbiota; *Mann-Whitney test with p value < 0.05.

(C–E) *Proteobacteria* (C), *Firmicutes* (D), and *Bacteroidetes* and *Tenericutes* (E) genus abundances of tissue-associated microbiota. All data in (C–E) have a p value < 0.05 (Mann-Whitney test with FDR corrected). Each dot represents individual mouse and bars show the means.

T3SS effectors are dedicated to dampening these innate immune processes (Pallett et al., 2017; Pearson et al., 2016).

Unexpectedly, alongside activation of cholesterol biosynthesis the cholesterol efflux transporters Abca1 and Abcg8 were also present at higher abundance in infected IECs. These transporters are likely functional, as significantly higher level of fecal cholesterol was detected in *C. rodentium*-infected mice. A previous study reported elevated levels of serum cholesterol during *C. rodentium* infection, although the reason for this was not apparent (Raczynski et al., 2012). Our study suggests that cholesterol efflux from IECs can reach the lumen of the gut via reverse cholesterol transport. Notably, increased fecal cholesterol was associated with a bloom of the colonic commensal *Serratia*, *Dickeya*, and *Erwinia*, which can metabolize cholesterol (Caspi et al., 2016; Garcia et al., 2012), and therefore benefit from the alteration of the gut niche which occurs as a consequence of *C. rodentium* (IEC interaction).

Under physiological conditions cholesterol limitation actives Srebp2 leading to cholesterol biogenesis and uptake, while an excess of cholesterol, which is cytotoxic, triggers expression of Abca1, Abcg5/8, and cholesterol efflux via the transcription regulator liver X receptor (Hong and Tontonoz, 2014). Using western blotting we found low levels of Abca1 and Abcg8 in IECs isolated from uninfected mice and robust expression in infected cells. Therefore, during *C. rodentium* infection both cholesterol biosynthesis and efflux are operating simultaneously. Importantly, at 14 DPI the abundance of the rate-limiting enzyme in the cholesterol biosynthetic pathway (hydroxymethylglutaryl-CoA reductase, Hmgcr) as well as Abca1, returned to pre-infection levels. Our data suggest that, while cholesterol biogenesis appears to be an innate immune IEC response to infection, the increased abundance of Abca1, Abcg5/8, ApoA1, and cholesterol efflux, concomitant with cholesterol production could represent yet another layer of defense *C. rodentium* erects while battling host immunity.

Taken together our data suggest that *C. rodentium* subverts metabolism in IECs to evade immune responses and change the oxygen availability at the apical surface of IECs. As IECs adapt to *C. rodentium*-induced disruption of the mitochondria by increasing glucose uptake, feeding glycolysis, and disseminating ATP via PCr, L-arginine is diverted from iNOS and NO production. Moreover, *C. rodentium* infection appears to dampen TLR4 signaling by triggering cholesterol efflux. As controlling the cholesterol circuit involves the pathogen, IECs, and inflammation, this phenotype has not been observed, or could not be easily studied, in cell culture models. Indeed, infection of

Caco-2 cells with EPEC impacts on central metabolism but does not induce the cholesterol biosynthetic pathway (Hardwidge et al., 2004).

Our data suggest that subversion of the central carbon metabolism in IECs is an important infection strategy, which is likely to be shared between *C. rodentium* and human pathogens. Therefore, our findings could open the way for development of new intervention strategies, either directly applied to the host, or indirectly via microbiome-based metabolite treatment (Suez and Elinav, 2017).

STAR★METHODS

Detailed methods are provided in the online version of this paper and include the following:

- KEY RESOURCES TABLE
- CONTACT FOR REAGENT AND RESOURCE SHARING
- EXPERIMENTAL MODEL AND SUBJECT DETAILS
  - Bacterial Strain
  - Animals
  - Cell Culture
- METHOD DETAILS
  - Generation of *C. rodentium Map* Mutant
  - Oral Gavage of Mice and CFU Count
  - Extraction of Enterocytes
  - Immunostaining of IECs
  - Tissue Staining and CCH Measurement
  - Bioluminescence Imaging
  - Fecal Cholesterol Measurement
  - Immunoblotting
  - [1-^{14}C] Sodium Butyrate Uptake Assay
  - Sample Preparation for TMT Labelling
  - Basic Reverse-Phase Peptide Fractionation
  - LC-ESI-MS/MS Analysis
  - Database Search and Protein Quantification
  - Bioinformatics Analysis
  - Lipid Fingerprint by MALDI-TOF MS
  - MALDI-TOF MS Analysis
  - Targeted Metabolomics of IECs
  - 16S rRNA Gene Sequencing
- QUANTIFICATION AND STATISTICAL ANALYSIS
  - Statistical Analysis
  - Quantification of BLI and Statistical Analyses
- DATA AND SOFTWARE AVAILABILITY

SUPPLEMENTAL INFORMATION

Supplemental Information includes five figures and four tables and can be found with this article online at https://doi.org/10.1016/j.cmet.2017.09.003.

AUTHOR CONTRIBUTIONS

V.F.C., D.C., and J.W.C. conducted the *in vivo* studies. C.N.B., V.F.C., T.I.R., J.C.W., L.Y., and J.S.C. analyzed the proteomics data. A.C. and R.C.D.F. conducted the butyrate uptake assay. T.I.R., J.C.W., L.Y., and J.S.C. performed the proteomic mass spectrometry experiments. M.P.-F., M.B., and E.E. profiled the microbiota. G.J.L.-M. performed the lipidomics and metabolomics assays. G.D., J.S.C., and G.F. provided supervision, guidance, and funding. C.N.B., V.F.C., T.I.R., J.C.W., L.Y., J.S.C., R.C.D.F., G.J.L.-M., and G.F. wrote the paper. C.N.B., V.F.C., T.I.R., and J.C.W. are joint first authors. They have contributed equally; the first two are based at Imperial College and were engaged in the *in vivo* work and data analysis, and the last two are based at the Sanger Institute and were responsible for the proteomics. G.F. and J.S.C. are joint corresponding authors. This work was done as a single project bringing together the biology of *C. rodentium* infection (headed by G.F.) and mass spectrometry (headed by J.S.C.). The complementary expertise and equal input into the study is reflected by a joint corresponding authorship.

ACKNOWLEDGMENTS

We thank Izabela Glegola-Madejska for an invaluable technical assistance in the *in vivo* experiments. E.E. is supported by: Y. and R. Ungar; the Leona M. and Harry B. Helmsley Charitable Trust; grants funded by the European Research Council; is the incumbent of the Rina Gudinski Career Development Chair, and is a senior fellow at the Canadian Institute for Advanced Research (CIFAR). J.C. and G.D. are supported by Wellcome Trust grant (WT098051) to the Sanger Institute. I.G-M. is supported by an MRC CMBI centre grant (MR/J006874/1). This project has been supported by an MRC program grant (MR/K019007/1) and a Wellcome investigator award (107057/Z/15/Z) to G.F.

Received: March 23, 2017
Revised: July 18, 2017
Accepted: September 6, 2017
Published: October 5, 2017

REFERENCES

Barker, N. (2014). Adult intestinal stem cells: critical drivers of epithelial homeostasis and regeneration. Nat. Rev. Mol. Cell Biol. *15*, 19–33.

Blachier, F., Beaumont, M., Andriamihaja, M., Davila, A.M., Lan, A., Grauso, M., Armand, L., Benamouzig, R., and Tome, D. (2017). Changes in the luminal environment of the colonic epithelial cells and physiopathological consequences. Am. J. Pathol. *187*, 476–486.

Borthakur, A., Gill, R.K., Hodges, K., Ramaswamy, K., Hecht, G., and Dudeja, P.K. (2006). Enteropathogenic *Escherichia coli* inhibits butyrate uptake in Caco-2 cells by altering the apical membrane MCT1 level. Am. J. Physiol. Gastrointest. Liver Physiol. *290*, G30–G35.

Caporaso, J.G., Kuczynski, J., Stombaugh, J., Bittinger, K., Bushman, F.D., Costello, E.K., Fierer, N., Pena, A.G., Goodrich, J.K., Gordon, J.I., et al. (2010). QIIME allows analysis of high-throughput community sequencing data. Nat. Methods *7*, 335–336.

Carlson-Banning, K.M., and Sperandio, V. (2016). Catabolite and oxygen regulation of enterohemorrhagic *Escherichia coli* virulence. MBio *7*, https://doi.org/10.1128/mBio.01852-16.

Caspi, R., Billington, R., Ferrer, L., Foerster, H., Fulcher, C.A., Keseler, I.M., Kothari, A., Krummenacker, M., Latendresse, M., Mueller, L.A., et al. (2016). The MetaCyc database of metabolic pathways and enzymes and the BioCyc collection of pathway/genome databases. Nucleic Acids Res. *44*, D471–D480.

Collins, J.W., Keeney, K.M., Crepin, V.F., Rathinam, V.A., Fitzgerald, K.A., Finlay, B.B., and Frankel, G. (2014). *Citrobacter rodentium*: infection, inflammation and the microbiota. Nat. Rev. Microbiol. *12*, 612–623.

Cox, J., and Mann, M. (2012). 1D and 2D annotation enrichment: a statistical method integrating quantitative proteomics with complementary high-throughput data. BMC Bioinformatics *13* (*Suppl 16*), S12.

Crepin, V.F., Habibzay, M., Glegola-Madejska, I., Guenot, M., Collins, J.W., and Frankel, G. (2015). Tir triggers expression of CXCL1 in enterocytes and neutrophil recruitment during *Citrobacter rodentium* infection. Infect. Immun. *83*, 3342–3354.

Donohoe, D.R., Garge, N., Zhang, X., Sun, W., O'Connell, T.M., Bunger, M.K., and Bultman, S.J. (2011). The microbiome and butyrate regulate energy metabolism and autophagy in the mammalian colon. Cell Metab. *13*, 517–526.

Fantin, V.R., St-Pierre, J., and Leder, P. (2006). Attenuation of LDH-A expression uncovers a link between glycolysis, mitochondrial physiology, and tumor maintenance. Cancer Cell *9*, 425–434.

Figurski, D.H., and Helinski, D.R. (1979). Replication of an origin-containing derivative of plasmid RK2 dependent on a plasmid function provided in trans. Proc. Natl. Acad. Sci. USA *76*, 1648–1652.

Fuchs, T.M., Eisenreich, W., Heesemann, J., and Goebel, W. (2012). Metabolic adaptation of human pathogenic and related nonpathogenic bacteria to extra- and intracellular habitats. FEMS Microbiol. Rev. *36*, 435–462.

Garcia, J.L., Uhia, I., and Galan, B. (2012). Catabolism and biotechnological applications of cholesterol degrading bacteria. Microb. Biotechnol. *5*, 679–699.

Ghisla, S., Hastings, J.W., Favaudon, V., and Lhoste, J.M. (1978). Structure of the oxygen adduct intermediate in the bacterial luciferase reaction: C nuclear magnetic resonance determination. Proc. Natl. Acad. Sci. USA *75*, 5860–5863.

Gierens, H., Nauck, M., Roth, M., Schinker, R., Schurmann, C., Scharnagl, H., Neuhaus, G., Wieland, H., and Marz, W. (2000). Interleukin-6 stimulates LDL receptor gene expression via activation of sterol-responsive and Sp1 binding elements. Arterioscler. Thromb. Vasc. Biol. *20*, 1777–1783.

Giogha, C., Lung, T.W., Pearson, J.S., and Hartland, E.L. (2014). Inhibition of death receptor signaling by bacterial gut pathogens. Cytokine Growth Factor Rev. *25*, 235–243.

Hardie, D.G. (2014). AMPK – sensing energy while talking to other signaling pathways. Cell Metab. *20*, 939–952.

Hardie, D.G., Ross, F.A., and Hawley, S.A. (2012). AMPK: a nutrient and energy sensor that maintains energy homeostasis. Nat. Rev. Mol. Cell Biol. *13*, 251–262.

Hardwidge, P.R., Rodriguez-Escudero, I., Goode, D., Donohoe, S., Eng, J., Goodlett, D.R., Aebersold, R., and Finlay, B.B. (2004). Proteomic analysis of the intestinal epithelial cell response to enteropathogenic *Escherichia coli*. J. Biol. Chem. *279*, 20127–20136.

Herrero, M., de Lorenzo, V., and Timmis, K.N. (1990). Transposon vectors containing non-antibiotic resistance selection markers for cloning and stable chromosomal insertion of foreign genes in gram-negative bacteria. J. Bacteriol. *172*, 6557–6567.

Hong, C., and Tontonoz, P. (2014). Liver X receptors in lipid metabolism: opportunities for drug discovery. Nat. Rev. Drug Discov. *13*, 433–444.

Huang, Z., Sutton, S.E., Wallenfang, A.J., Orchard, R.C., Wu, X., Feng, Y., Chai, J., and Alto, N.M. (2009). Structural insights into host GTPase isoform selection by a family of bacterial GEF mimics. Nat. Struct. Mol. Biol. *16*, 853–860.

Jones, R.G., Plas, D.R., Kubek, S., Buzzai, M., Mu, J., Xu, Y., Birnbaum, M.J., and Thompson, C.B. (2005). AMP-activated protein kinase induces a p53-dependent metabolic checkpoint. Mol. Cell *18*, 283–293.

Joseph, A.M., Pilegaard, H., Litvintsev, A., Leick, L., and Hood, D.A. (2006). Control of gene expression and mitochondrial biogenesis in the muscular adaptation to endurance exercise. Essays Biochem. *42*, 13–29.

Joseph, J., Cardesa, A., and Carreras, J. (1997). Creatine kinase activity and isoenzymes in lung, colon and liver carcinomas. Br. J. Cancer *76*, 600–605.

Kaniga, K., Delor, I., and Cornelis, G.R. (1991). A wide-host-range suicide vector for improving reverse genetics in gram-negative bacteria: inactivation of the blaA gene of *Yersinia enterocolitica*. Gene *109*, 137–141.

Kilkenny, C., Browne, W.J., Cuthill, I.C., Emerson, M., and Altman, D.G. (2010). Improving bioscience research reporting: the ARRIVE guidelines for reporting animal research. PLoS Biol. *8*, e1000412.

Koppenol, W.H., Bounds, P.L., and Dang, C.V. (2011). Otto Warburg's contributions to current concepts of cancer metabolism. Nat. Rev. Cancer *11*, 325–337.

Kvandova, M., Majzunova, M., and Dovinova, I. (2016). The role of PPARgamma in cardiovascular diseases. Physiol. Res. *65*, S343–S363.

Lahiri, A., Das, P., and Chakravortty, D. (2008). Arginase modulates *Salmonella* induced nitric oxide production in RAW264.7 macrophages and is required for *Salmonella* pathogenesis in mice model of infection. Microbes Infect. *10*, 1166–1174.

Lopez, C.A., Miller, B.M., Rivera-Chavez, F., Velazquez, E.M., Byndloss, M.X., Chavez-Arroyo, A., Lokken, K.L., Tsolis, R.M., Winter, S.E., and Baumler, A.J. (2016). Virulence factors enhance *Citrobacter rodentium* expansion through aerobic respiration. Science *353*, 1249–1253.

Ma, C., Wickham, M.E., Guttman, J.A., Deng, W., Walker, J., Madsen, K.L., Jacobson, K., Vogl, W.A., Finlay, B.B., and Vallance, B.A. (2006). *Citrobacter rodentium* infection causes both mitochondrial dysfunction and intestinal epithelial barrier disruption in vivo: role of mitochondrial associated protein (Map). Cell Microbiol. *8*, 1669–1686.

Marteyn, B., West, N.P., Browning, D.F., Cole, J.A., Shaw, J.G., Palm, F., Mounier, J., Prevost, M.C., Sansonetti, P., and Tang, C.M. (2010). Modulation of *Shigella* virulence in response to available oxygen in vivo. Nature *465*, 355–358.

Miliara, X., Garnett, J.A., Tatsuta, T., Abid Ali, F., Baldie, H., Perez-Dorado, I., Simpson, P., Yague, E., Langer, T., and Matthews, S. (2015). Structural insight into the TRIAP1/PRELI-like domain family of mitochondrial phospholipid transfer complexes. EMBO Rep. *16*, 824–835.

Mundy, R., MacDonald, T.T., Dougan, G., Frankel, G., and Wiles, S. (2005). *Citrobacter rodentium* of mice and man. Cell Microbiol. *7*, 1697–1706.

Mundy, R., Schuller, S., Girard, F., Fairbrother, J.M., Phillips, A.D., and Frankel, G. (2007). Functional studies of intimin in vivo and ex vivo: implications for host specificity and tissue tropism. Microbiology *153*, 959–967.

Neis, E.P., Dejong, C.H., and Rensen, S.S. (2015). The role of microbial amino acid metabolism in host metabolism. Nutrients *7*, 2930–2946.

Pallett, M.A., Crepin, V.F., Serafini, N., Habibzay, M., Kotik, O., Sanchez-Garrido, J., Di Santo, J.P., Shenoy, A.R., Berger, C.N., and Frankel, G. (2017). Bacterial virulence factor inhibits caspase-4/11 activation in intestinal epithelial cells. Mucosal Immunol. *10*, 602–612.

Paradies, G., Paradies, V., De Benedictis, V., Ruggiero, F.M., and Petrosillo, G. (2014). Functional role of cardiolipin in mitochondrial bioenergetics. Biochim. Biophys. Acta *1837*, 408–417.

Pearson, J.S., Giogha, C., Wong Fok Lung, T., and Hartland, E.L. (2016). The genetics of enteropathogenic *Escherichia coli* virulence. Annu. Rev. Genet. *50*, 493–513.

Peterson, L.W., and Artis, D. (2014). Intestinal epithelial cells: regulators of barrier function and immune homeostasis. Nat. Rev. Immunol. *14*, 141–153.

Raczynski, A.R., Muthupalani, S., Schlieper, K., Fox, J.G., Tannenbaum, S.R., and Schauer, D.B. (2012). Enteric infection with *Citrobacter rodentium* induces coagulative liver necrosis and hepatic inflammation prior to peak infection and colonic disease. PLoS One *7*, e33099.

Ren, M., Phoon, C.K., and Schlame, M. (2014). Metabolism and function of mitochondrial cardiolipin. Prog. Lipid Res. *55*, 1–16.

Ruano-Gallego, D., Alvarez, B., and Fernandez, L.A. (2015). Engineering the controlled assembly of filamentous injectisomes in *E. coli* K-12 for protein translocation into mammalian cells. ACS Synth. Biol. *4*, 1030–1041.

Schauer, D.B., and Falkow, S. (1993). Attaching and effacing locus of a *Citrobacter freundii* biotype that causes transmissible murine colonic hyperplasia. Infect. Immun. *61*, 2486–2492.

Schiering, C., Wincent, E., Metidji, A., Iseppon, A., Li, Y., Potocnik, A.J., Omenetti, S., Henderson, C.J., Wolf, C.R., Nebert, D.W., et al. (2017). Feedback control of AHR signalling regulates intestinal immunity. Nature *542*, 242–245.

Southan, G.J., Szabo, C., and Thiemermann, C. (1994). Inhibition of the induction of nitric oxide synthase by spermine is modulated by aldehyde dehydrogenase. Biochem. Biophys. Res. Commun. *203*, 1638–1644.

Spann, N.J., and Glass, C.K. (2013). Sterols and oxysterols in immune cell function. Nat. Immunol. *14*, 893–900.

Suez, J., and Elinav, E. (2017). The path towards microbiome-based metabolite treatment. Nat. Microbiol. *2*, 17075.

Tall, A.R., and Yvan-Charvet, L. (2015). Cholesterol, inflammation and innate immunity. Nat. Rev. Immunol. *15*, 104–116.

Tyanova, S., Temu, T., Sinitcyn, P., Carlson, A., Hein, M.Y., Geiger, T., Mann, M., and Cox, J. (2016). The Perseus computational platform for comprehensive analysis of (prote)omics data. Nat. Methods *13*, 731–740.

Vallance, B.A., Deng, W., De Grado, M., Chan, C., Jacobson, K., and Finlay, B.B. (2002). Modulation of inducible nitric oxide synthase expression by the attaching and effacing bacterial pathogen *Citrobacter rodentium* in infected mice. Infect. Immun. *70*, 6424–6435.

Wallimann, T., Tokarska-Schlattner, M., and Schlattner, U. (2011). The creatine kinase system and pleiotropic effects of creatine. Amino acids *40*, 1271–1296.

Wiles, S., Clare, S., Harker, J., Huett, A., Young, D., Dougan, G., and Frankel, G. (2004). Organ specificity, colonization and clearance dynamics in vivo following oral challenges with the murine pathogen *Citrobacter rodentium*. Cell Microbiol. *6*, 963–972.

Wolfgang, M.J., and Lane, M.D. (2006). The role of hypothalamic malonyl-CoA in energy homeostasis. J. Biol. Chem. *281*, 37265–37269.

Wong, A.R., Pearson, J.S., Bright, M.D., Munera, D., Robinson, K.S., Lee, S.F., Frankel, G., and Hartland, E.L. (2011). Enteropathogenic and enterohaemorrhagic *Escherichia coli*: even more subversive elements. Mol. Microbiol. *80*, 1420–1438.

Yu, F., He, F., Yao, H., Wang, C., Wang, J., Li, J., Qi, X., Xue, H., Ding, J., and Zhang, P. (2015). Structural basis of intramitochondrial phosphatidic acid transport mediated by Ups1-Mdm35 complex. EMBO Rep. *16*, 813–823.

STAR★METHODS

KEY RESOURCES TABLE

REAGENT or RESOURCE	SOURCE	IDENTIFIER
**Antibodies**		
Ki-67, rabbit monoclonal antibody (Clone SP6)	ThermoFisher Scientific	Cat# RM-9106-F0; RRID: AB_721371
E-Cadherin, purified mouse antibody (Clone 36)	BD Transduction Laboratories™	Cat# 610182; RRID: AB_397581
Intimin β, purified chicken antibody IgY	John Morris Fairbrother – (Mundy et al., 2007)	N/A
O152, Rabbit Polyclonal Antibody	Claire Jenkins, Public Health England –(Schiering et al., 2017)	N/A
Phospho-AMPKα (Thr172), purified rabbit monoclonal antibody (Clone 40H9)	Cell Signaling Technology	Cat# 2535; RRID: AB_331250
Gapdh, rabbit polyclonal antibody	Abcam	Cat# ab9485; RRID: AB_307275
Srebp2, rabbit polyclonal antibody	Abcam	Cat# ab30682; RRID: AB_779079
Abca1, mouse monoclonal antibody (clone AB.H10)	Abcam	Cat# ab18180; RRID: AB_444302
Abcg8, rabbit polyclonal antibody	Abcam	Cat# ab126493; RRID: AB_11130138
Cy™3 AffiniPure Goat Anti-Chicken IgY (IgG) (H+L)	Jackson ImmunoResearch	Cat# 103-005-155; RRID: AB_2337379
AMCA AffiniPure Donkey Anti-Mouse IgG (H+L)	Jackson ImmunoResearch	Cat# 715-155-150; RRID: AB_2340806
Alexa Fluor® 488 AffiniPure Donkey Anti-Rabbit IgG (H+L)	Jackson ImmunoResearch	Cat# 711-545-152; RRID: AB_2313584
Peroxidase AffiniPure Goat Anti-Rabbit IgG, Fc fragment specific	Jackson ImmunoResearch	Cat# 111-035-008; RRID: AB_2337937
Peroxidase AffiniPure Goat Anti-Mouse IgG, Fcγ fragment specific	Jackson ImmunoResearch	Cat# 115-035-008; RRID: AB_2313585
**Bacterial and Virus Strains**		
Wild type *C. rodentium*	Pr. Frankel, (Schauer and Falkow, 1993)	ICC169
C. rodentium ICC169::*Lux*	Pr. Frankel, (Wiles et al., 2004)	ICC180
C. rodentium ICC180 Δ*map*	This study	ICC1411
C. rodentium ICC180 Δ*map*::*map*	This study	ICC1412
pSEVA612S-map	Pr. Frankel, Imperial College	pICC2536
pSEVA612S-HR	Pr. Frankel, Imperial College	pICC2537
pACBSR	Ruano-Gallego et al., 2015	N/A
pRK2013	Figurski and Helinski, 1979	N/A
**Chemicals, Peptides, and Recombinant Proteins**		
Phalloidin–Tetramethylrhodamine B isothiocyanate	Sigma-Aldrich	P1951-.1MG
C^{14} -Sodium butyrate	American Radiochemicals Inc.	ARC 0191-250
**Critical Commercial Assays**		
Total Cholesterol Assay Kits	Cambridge bioscience	STA-384
**Deposited Data**		
Mass spectrometry proteomics data	ProteomeXchange Consortium (PRIDE)	PXD005004
**Experimental Models: Cell Lines**		
Caco-2 - TC7 clone (male)	Pr. Imad Kansau (Universite Paris Sud)	N/A
**Experimental Models: Organisms/Strains**		
Pathogen-free female C57BL/6 mice	Charles River, UK	Strain Code: 027
Pathogen-free female C3H/HeNCrl mice	Charles River, UK	Strain Code: 025

(*Continued on next page*)

Continued		
REAGENT or RESOURCE	SOURCE	IDENTIFIER
Oligonucleotides		
TACTGCATGCTGTGCAAGATCTGTGAGAAA TTGTTCATTCAT	Pr. Frankel, Imperial College	DC074
TACTGAGCTCTTTATATTGTTATGATGCAA CGGTATGCAGTC	Pr. Frankel, Imperial College	DC075
ATAGAAAAAACATACCAAGCATTTCTCGGT	Pr. Frankel, Imperial College	DC084
CAGGGGAGAAAATAATAAACGAGATCC	Pr. Frankel, Imperial College	DC085
Software and Algorithms		
Ingenuity® Pathway Analysis (IPA®)	Qiagen	https://www.qiagen.com/dk/products/life-science-research/research-applications/gene-expression-analysis/analysis/ingenuity-pathway-analysis/
Living Image Software 4.3.1	Perkin Elmer	http://www.perkinelmer.co.uk/product/lumina-kinetic-xr-100-living-image-v4se-128110
Proteome Discoverer™ Software	ThermoFisher Scientific	https://www.thermofisher.com/order/catalog/product/IQLAAEGABSFAKJMAUH
Perseus 1.4	Max Planck Institute of Biochemistry	http://www.coxdocs.org/doku.php?id=perseus:start
QIIME	(Caporaso et al., 2010)	http://qiime.org/
Data Explorer 4.9	Applied Biosystems	N/A

CONTACT FOR REAGENT AND RESOURCE SHARING

Further information and requests for resources and reagents should be directed to and will be fulfilled by the Lead Contact, Gad Frankel (g.frankel@imperial.ac.uk).

EXPERIMENTAL MODEL AND SUBJECT DETAILS

Bacterial Strain

C. rodentium strains listed in Table S1 were grown at 37°C in Luria–Bertani (LB) with necessary antibiotics as indicated in Tables S1 and S2 at the following concentrations: nalidixic acid (50 μg/ml), kanamycin (50 μg/ml), streptomycin (50 μg/ml) or gentamicin (10 μg/ml).

Animals

All animal experiments were performed in accordance with the Animals Scientific Procedures Act 1986 and were approved by the local Ethical Review Committee and UK Home office guidelines. Experiments were designed in agreement with the ARRIVE guidelines (Kilkenny et al., 2010), for the reporting and execution of animal experiments, including sample randomization and blinding. Pathogen-free female C57BL/6 mice (18 to 20 g) or C3H/HeNCrl mice (18 to 24 g) were purchased from Charles River, UK. All mice were housed in individually HEPA-filtered cages with sterile bedding (Processed corncobs grade 6), nesting (LBS Serving technology) and free access to sterilized food (LBS Serving technology) and water. A minimum of 4 and a maximum of 8 mice randomly assigned for each group were used per experiment. Each experiment was repeated a minimum of two times.

Cell Culture

Human (male) epithelial colorectal adenocarcinoma cells (Caco-2), clone TC-7 were maintained in DMEM with glucose (1g/L; Sigma) supplemented with 20% (v/v) FCS (Gibco), 2 mM Glutamax (Sigma) and 0.1 mM non-essential amino acids (NEAA) (Sigma) and incubated at 37°C, 10% CO_2.

METHOD DETAILS

Generation of *C. rodentium Map* Mutant

All plasmids and primers used are listed in Tables S2 and S3, respectively. The *map* flanking regions were synthetized by GeneArt (ThermoFisher) and sub-cloned into pSEVA612S vector. Alternatively, *map* and its flanking regions were PCR amplified (primer DC074 and DC075) from purified *C. rodentium* genomic DNA. The PCR amplicon was purify using a PCR purification kit (Qiagen), digested in CutSmart buffer at 37°C for 2 hours with the High Fidelity Enzymes SacI and SphI (New England Biolabs) and ligated

into pSEVA612S vector using T4 Ligase (New Engand Biolabs) for 2 hours at room temperature. The pSEVA612 derivatives were then chemically transformed in CC118λpir *E. coli* (Herrero et al., 1990).

The *map* gene was deleted from *C. rodentium* ICC180 pre-transformed with pACBSR – a plasmid containing the endonuclease I-SceI (Ruano-Gallego et al., 2015), using tri-parental conjugation. Donor strain (*E. coli* CC118λpir containing pSEVA612 derivatives), helper strain (*E. coli* CC1047 (Kaniga et al., 1991), containing pRK2013 (Figurski and Helinski, 1979)), and ICC180-pACBSR were combined and grown for at least 6 h prior to overnight selection on LB agar supplemented with gentamicin and spectinomycin. Selected colonies were grown in LB broth supplemented with and L-arabinose (0.4%) for a minimum of 6 h, to induce the I-SceI endonuclease from pACBSR plasmid. Cultures were subsequently streaked out for overnight growth on LB spectinomycin. Colonies were screened by PCR for successful *map* deletion, using primers DC084 and DC085. The same method was used for re-insertion of *map* onto the genome. *C. rodentium* strains were sequenced (GATC Biotech) to confirm deletion and re-insertion of *map*.

Oral Gavage of Mice and CFU Count

Mice were inoculated by oral gavage with 200 μl of overnight LB-grown *C. rodentium* suspension 10X concentrated in PBS (~5 x10^9 colony forming units (cfu)). Uninfected mice were mock treated with PBS (200 μl). The number of viable bacteria used as inoculum was determined by retrospective plating onto LB agar containing nalidixic acid. Stool samples were recovered at regular intervals after inoculation and the number of viable bacteria per gram of stool was determined by plating onto LB agar containing nalidixic acid. For determining tissue associated CFU, 4 cm of distal colonic tissues were harvested, opened longitudinally to allow stools removals, washed in PBS and homogenized in 10 ml PBS per gram of tissue using an gentleMACs automated tissue dissociator (Miltenyi Biotech). The aqueous layer was plated on LB agar containing nalidixic acid and the CFU were quantified.

Extraction of Enterocytes

At 8 DPI, 4-cm segment of terminal colon was cut longitudinally, placed in 4 ml enterocyte dissociation buffer (1X Hanks' balanced salt solution without Mg and Ca, containing 10 mM HEPES, 1 mM EDTA and 5 μl/ml 2-β-mercaptoethanol), and incubated at 37°C with shaking, for 45 min. The enterocytes were collected by centrifugation (2,000 x *g* for 10 min) followed by two PBS washes. Enterocytes pellets were either kept frozen for proteomic analysis and Western blotting or fixed in 4% formaldehyde for immunofluorescence staining.

Immunostaining of IECs

Fixed enterocytes were permeabilized with 0.1% Triton and stained with rabbit polyclonal anti O152 antiserum (a gift from Claire Jenkins, Public Health England) for 20 min, followed by 30 min of incubation with standard secondary as described above and with Phalloidin-TRITC (Sigma) to visualize actin filament. Samples were analyzed with an Axio Imager M1 microscope (Carl Zeiss MicroImaging GmbH, Germany), and images were acquired using an AxioCam MRm monochrome camera and computer processed using AxioVision (Carl Zeiss MicroImaging GmbH, Germany).

Tissue Staining and CCH Measurement

Half a centimeter of terminal colon of each mouse was collected, flushed with PBS and fixed in 1 ml 10% neutral buffered formalin. Formalin fixed tissues were then processed, paraffin-embedded and sectioned at 5μm. Paraffin-embedded sections (FFPE) were either stained with haematoxylin and eosin (H&E) using standard techniques or treated with sodium citrate antigen de-masking solution prior to immunofluorescence. Primary antibodies were used at 1:200 dilution for anti-intimin (a gift from Professor Fairbrother, Montreal University) and 1:50 for E-cadherin (CD324; BD Biosciences) and Ki67 (SP6; Thermo Scientific) followed by secondary antibodies from Jackson ImmunoResearch used at a 1:200 dilution (donkey anti chicken Cy3, donkey anti mouse AMCA, donkey anti rabbit – AlexaFluor 488). H&E stained tissues were evaluated blindly for CCH microscopically by measuring the length of at least 20 well-oriented crypts from each section from all of the mice per treatment group. Similarly, Ki-67 staining was assessed microscopically by measuring the distance from the bottom of the crypt to the last stained nuclei. For comparison, Ki-67staining was expressed as a ratio over the total length of the crypt. Tissues were imaged with an Axio, images were acquired using an Axio camera, and computer-processed using AxioVision (Carl Zeiss MicroImaging GmbH, Germany).

Bioluminescence Imaging

For bioluminescent imaging (BLI), C3H/HeNCrl mice were depilated using hair removal cream (Veet) prior to infection to remove pigmented fur that may interfere with signal output. At 6 DPI, animals were imaged using the IVIS® Spectrum CT (Perkin Elmer) system under gaseous anesthesia with isofluorane (Zoetis).

Fecal Cholesterol Measurement

Total fecal cholesterol (cholesterol esters and free cholesterol) was quantified using a colorimetric reaction, as per manufacturer recommendations (Cell Biolabs, STA-384). Stools were harvested from uninfected and infected mice 8 DPI, vacuum dried and weighed before being crunched to powder and extracted in 800 μl of a mixture of chloroform : isopropanol : NP-40 (7:11:0.1). The colorimetric signal was analyzed using a spectrophotometric microplate reader in the 540-570 nm range.

Immunoblotting

Proteins were resolved by SDS-PAGE, and gels were transferred to polyvinylidene difluoride membrane (GE Healthcare). Membranes were washed with TBS 0.2% Tween, blocked in TBS supplemented with 0.2% Tween, 3% BSA and 0.5% gelatin for 30 min at room temperature and probed with specific antibodies overnight at 4°C. After three washes of 10 min in TBS 0.2% Tween, blots were incubated with horseradish peroxidase-linked secondary antibody (Jackson ImmunoResearch) for 45 min at room temperature, followed by EZ-ECL assay, according to the manufacturer's instructions (Biological Industries). Chemiluminescence was detected using a Chemidoc (BioRad). Primary antibodies used were: anti-Srebp2 (ab30682; Abcam), p-AMPKα (#2535; cell signaling), Abca1 (ab18180; Abcam), Abcg8 (ab126493; Abcam) and anti-Gapdh (ab9485; Abcam).

[1-^{14}C] Sodium Butyrate Uptake Assay

Caco-2/TC-7 cells were seeded at 3.5×10^4 in 1 ml of media in triplicate in 24-well plates 12-14 days prior to the experiment and the media replaced every 48 hours. 4. Caco-2 media was changed for serum free media the night before infection (1g/L glucose DMEM with NEAA and glutamax). The day before the experiment, *C. rodentium* was grown for eight hours during the day in LB, before dilution 1:100 in DMEM (1g/L glucose) (no supplements) and incubation over night at 37°C, 10% CO2. The morning of the infection, *C. rodentium* cultures were diluted 1:10 into fresh DMEM and Caco-2's infected with 5×10^7 bacteria per well. Plates were centrifuged at 100 x*g* for 5 minutes to synchronize infections before incubation for 2.5 hours. After infection, cells were washed 1 x PBS and incubated in HBSS supplemented with 1.3 mM $CaCl_2$, 5.4 mM KCl, 0.44 mM K_2HPO_4, 0.4 mM $MgSO_4$, 0.4 mM Na_2HPO_4, 4mM $NaHCO_3$, 0.5 mM $MgCl_2$, 135 mM choline chloride, and 10 mM HEPES pH 7.5 for 15 minutes. Cells were washed 1 x HBSS pH 6.5 and 0.2 mM [1-^{14}C] Sodium Butyrate (American Radiochemicals Inc.) added for 5 minutes. After incubation, cells were washed 3 x ice cold HBSS pH 6.5 before solubilisation in 0.1 M NaOH/0.1% SDS for 4 hours at 37°C. Protein concentration was measured using a BCA assay (Pierce) and incorporated radioactivity was counted using a Wallac 1409 DSA liquid scintillation counter, in conjunction with Ultima Gold™ scintillation fluid (Perkin Elmer).

Sample Preparation for TMT Labelling

IEC pellets, isolated from *C. rodentium* infected and uninfected mice, were dissolved in 100 μL 0.1 M triethylammonium bicarbonate (TEAB), 0.1% SDS assisted with pulsed probe sonication. Protein concentration was measured with Quick Start Bradford Protein Assay (Bio-Rad) according to manufacturer's instructions. Aliquots containing 100 μg of total protein were prepared for trypsin digestion. Samples were reduced with tris-2-carboxymethyl phosphine (TCEP) and alkylated with Iodoacetamide (IAA), followed by trypsin digest as described above. The resultant peptides were diluted up to 100 μL with 0.1 M TEAB buffer and labelled with TMT 10-plex reagent vial (Thermo Scientific) according to manufactures instructions.

Basic Reverse-Phase Peptide Fractionation

Offline peptide fractionation based on high pH Reverse Phase (RP) chromatography was performed using the Waters, XBridge C18 column (2.1 x 150 mm, 3.5 μm, 120 Å) on a Dionex Ultimate 3000 HPLC system equipped with an auto sampler. Mobile phase A was composed of 0.1% ammonium hydroxide and mobile phase B was composed of 100% acetonitrile, 0.1% ammonium hydroxide. The TMT labelled peptide mixture was reconstituted in 100 μL mobile phase A, for fractionation using a multi-step gradient elution method at 0.2 mL/min as follows: for 5 minutes isocratic at 5% B, for 35 min gradient to 35% B, gradient to 80% B in 5 min, isocratic for 5 minutes and re-equilibration to 5% B. Fractions were collected in a time dependent manner every 30 sec and dried.

LC-ESI-MS/MS Analysis

LC-MS analysis was performed on the Dionex Ultimate 3000 UHPLC system coupled with the Orbitrap Fusion Tribrid Mass Spectrometer (Thermo Scientific). Each peptide fraction was reconstituted in 40 μL 0.1% formic acid and a volume of 7 μL was loaded to the Acclaim PepMap 100, 100 μm × 2 cm C18, 5 μm, 100 Å trapping column with the μlPickUp mode at 10 μL/min flow rate. The sample was then subjected to a multi-step gradient elution on the Acclaim PepMap RSLC (75 μm × 50 cm, 2 μm, 100 E) C18 capillary column (Dionex) retrofitted to an electrospray emitter (New Objective, FS360-20-10-D-20) at 45 °C. Mobile phase A was composed of 100% H_2O, 0.1% formic acid and mobile phase B was composed of 80% acetonitrile, 0.1% formic acid. The gradient separation method at flow rate 300 nL/min was as follows: for 95 min gradient to 42% B, for 5 min up to 95% B, for 8 min isocratic at 95% B, re-equilibration to 5% B in 2 min, for 10 min isocratic at 5% B. Precursors between 400-1500 *m/z* were selected with mass resolution of 120 k, AGC 3×10^5 and IT 100 ms with the top speed mode in 3 sec and were isolated for CID fragmentation with quadrupole isolation width 0.7 Th. Collision energy was set at 35% with AGC 1×10^4 and IT 35 ms. MS3 quantification spectra were acquired with further HCD fragmentation of the top 10 most abundant CID fragments isolated with Synchronous Precursor Selection (SPS) excluding neutral losses of maximum m/z 30. Quadrupole isolation width was set at 0.5 Th, collision energy was applied at 45% and the AGC setting was at 6×10^4 with 100ms IT. The HCD MS3 spectra were acquired only for the mass range 120-140 with 60k resolution. Targeted precursors were dynamically excluded for further isolation and activation for 45 seconds with 7 ppm mass tolerance.

Database Search and Protein Quantification

The acquired mass spectra were submitted to the SequestHT search engine implemented in the Proteome Discoverer 2.1 (Thermo Scientific) software for protein identification and quantification. The precursor mass tolerance was set at 20 ppm and the fragment ion

mass tolerance was set at 0.5 Da. Spectra were searched for fully tryptic peptides with maximum 2 miss-cleavages and minimum length of 6 amino acids. TMT6plex at N-terminus, K and Carbamidomethyl at C were defined as static modifications. Dynamic modifications included oxidation of M and Deamidation of N, Q. A maximum of two different dynamic modifications were allowed for each peptide. Peptide confidence was estimated with the Percolator node. Peptide FDR was set at 0.01 and validation was based on p-value and decoy database search. All spectra were searched against a fasta files containing the UniProt Reference Proteomes of 16,608 mouse reviewed protein entries. The reporter ion quantifier node included a custom TMT 10plex quantification method with an integration window tolerance of 15 ppm and integration method based on the most confident centroid peak at the MS3 level. Only unique peptides were used for quantification, considering protein groups for peptide uniqueness. Peptides with average reported S/N>3 were used for protein quantification. The enterocytes obtained from uninfected mice were used as controls for log2 ratio calculations. Differential expression p-values were computed based on a single-sample t-test using the Perseus proteomics tool and the average protein log2 ratios from the two measurements in each time point were used for downstream analyses. Specificity thresholds used for the analysis were defined as log p-value > 0.697 and log2 ratio > 0.59 or < -0.59 (equivalent to 1.5 fold change).

Bioinformatics Analysis

Differential protein abundance p-values were computed based on one-sample t-test using the Perseus 1.4 software (Tyanova et al., 2016) and the respective volcano plot was drawn in R using the ggplot2 package. KEGG pathway enrichment analysis was performed in Perseus 1.4 software (Tyanova et al., 2016) with the 1D-annotation enrichment method (Cox and Mann, 2012). The enrichment score indicates whether the proteins in a given pathway tend to be systematically up-regulated (positive score) or down-regulated (negative score) based on a Wilcoxon-Mann-Whitney test. Significantly enriched KEGG pathways were filtered for Benjamini-Hochberg FDR<0.05.

Specifically regulated protein 8 DPI were upload in Ingenuity Pathway Analysis (IPA) (Qiagen) platform which provides a comprehensive knowledgebase of curated experimentally observed annotations as well as reviewed findings from third party resources. Statistically significant over-representation of canonical pathways, cellular and molecular functions and enrichment of upstream regulators were calculated using the right-tailed Fisher Exact Test. The Benjamini-Hochberg method was used for multiple testing corrections (p<0.05). Trends of activation/inhibition states of the enriched functions and regulators were inferred by the calculation of a z-score (-2 < z-score > 2). IPA was used for construction and visualization of interaction networks using experimentally observed relationships that included direct or indirect interactions.

Lipid Fingerprint by MALDI-TOF MS

Isolated IECs or bacterial culture were washed three times with 0.5 ml of double distilled water at 15,000xg for 5 min and the supernatant was discard. The pellet was suspended in double distilled water. Prior to mass spectrometry analysis, the 2, 5-dihydroxybenzoic acid (DHB) matrix was used at a concentration of 10 mg/ml in chloroform/methanol 90:10 v/v. 0.4 μl of biological sample and 0.8 μl of the matrix solution were deposited on the target, mixed with a micropipette and dried under a gentle stream of air.

MALDI-TOF MS Analysis

MALDI-TOF MS analysis was performed on a 4800 Proteomics Analyzer (with TOF-TOF Optics, Applied Biosystems) using the reflectron mode. Samples were analyzed operating at 20 kV in the negative ion mode using an extraction delay time set at 20 ns. Typically, spectra from 500 to 2,000 laser shots were summed to obtain the final spectrum. All experiments were carried out in three independent biological repeats. Mass spectrometry data were analyzed using Data Explorer version 4.9 from Applied Biosystems.

Targeted Metabolomics of IECs

Isolated IECs were suspended in PBS and metabolically quenched by addition of acetonitrile/methanol/H_2O (2:2:1) on ice. Metabolites were extracted by mechanical lysing with a micropipette. Lysates were clarified by centrifugation and filtered through 0.22 μm Spin-X column filters (Costar®). Biomass of individual samples was determined by measuring the residual protein content of the metabolite extracts using the BCA assays kit (Thermo®). Aqueous normal phase liquid chromatography was performed using an Agilent 1290 Infinity II LC system equipped with a binary pump, temperature-controlled auto-sampler (set at 4°C) and temperature-controlled column compartment (set at 25°C), containing a Cogent Diamond Hydride Type C silica column (150 mm × 2.1 mm; dead volume 315 μl). A flow-rate of 0.4 ml/min was used. Elution of polar metabolites was carried out using solvent A consisting of deionized water (Resistivity ~ 18 MΩ cm), 0.2% acetic acid and solvent B consisting of acetonitrile and 0.2% acetic acid in acetonitrile. The following gradient was used: 0 min 85% B; 0-2 min 85% B; 3-5 min to 80% B; 6-7 min 75% B; 8-9 min 70% B; 10-11 min 50% B; 11.1-14 min 20% B; 14.1-25 min hold 20% B follow by a 5 min re-equilibration period in 85% B at a flow-rate of 0.4 ml/min. Accurate mass spectrometry was carried out using an Agilent Accurate Mass 6545 QTOF apparatus. Dynamic mass axis calibration was achieved by continuous infusion, post-chromatography, of a reference mass solution using an isocratic pump connected to an ESI ionization source, operated in the positive-ion mode. Nozzle Voltage and fragmentor voltages were set at 2,000 V and 100 V, respectively. The nebulizer pressure was set at 50 psi and the nitrogen drying gas flow rate was set at 5 l/min. The drying gas temperature was maintained at 300°C. The MS acquisition rate was 1.5 spectra/sec and m/z data ranging from 50-1,200 were stored. This instrument routinely enabled accurate mass spectral measurements with an error of less than 5 parts-per-million (ppm), mass

resolution ranging from 10,000-25,000 over the m/z range of 121-955 atomic mass units, and a 100,000-fold dynamic range with picomolar sensitivity. The data were collected in the centroid mode in the 4 GHz (extended dynamic range) mode.

16S rRNA Gene Sequencing

Colons were collected from mice and DNA was isolated using PowerSoil DNA Isolation Kit (MO BIO Laboratories). For 16S amplicon pyrosequencing, PCR amplification was performed spanning the V3and V4 region using the primers 515F/806R of the 16S rRNA gene and subsequently sequenced using 500bp paired-end sequencing (Illumina MiSeq). Reads were then processed using the QIIME (quantitative insights into microbial ecology) analysis pipeline with USEARCH against the Greengenes database. Importantly, the *C. rodentium* 16SRNA sequence was recognized by the Greengene database as *Enterobacter*, as the two differ in only 8 bp (hence the combined classification in Figure 7C).

QUANTIFICATION AND STATISTICAL ANALYSIS

Statistical Analysis

GraphPad Prism software was used for all statistical calculations. Statistical test used was Mann-Whitney compared to controls (or as indicated in the figure). P-values < 0.05 were considered significant. For the microbiota, p-values were FDR corrected using Benjamini and Hochberg method.

Quantification of BLI and Statistical Analyses

Analysis of IVIS® Spectrum images was carried out on Living image software. Photons from regions of interest (ROI) of a defined size (3.5 x 5cm) were quantified as total photon flux (p/s). All statistical analysis was carried out using GraphPad Prism 7.0. A multiple t-test was used to identify statistical significance for total flux output of bioluminescent images.

DATA AND SOFTWARE AVAILABILITY

The mass spectrometry proteomics data have been deposited to the ProteomeXchange Consortium via the PRIDE partner repository with the dataset identifier PXD005004.

Cell Metabolism, Volume *26*

Supplemental Information

Citrobacter rodentium Subverts ATP Flux and Cholesterol Homeostasis in Intestinal Epithelial Cells *In Vivo*

Cedric N. Berger, Valerie F. Crepin, Theodoros I. Roumeliotis, James C. Wright, Danielle Carson, Meirav Pevsner-Fischer, R. Christopher D. Furniss, Gordon Dougan, Mally Dori-Bachash, Lu Yu, Abigail Clements, James W. Collins, Eran Elinav, Gerald J. Larrouy-Maumus, Jyoti S. Choudhary, and Gad Frankel

Supplementary figure and tables

Figure S1. Relative abundance of metabolic proteins (related to Figure 2A, 2D, 3A, 5A, 5B, 6A and 6C).

Bar plots showing the relative abundance of the individual proteins: **A.** mitochondrial membrane proteins (related to Figure 2A), **B.** TCA cycle (related to Figure 2D), **C.** cardiolipin biosynthesis (related to Figure 3A), **D.** Glycolysis (related to Figure 5A), **E.** creatine biosynthesis (related to Figure 5B), **F.** AMPK pathway (related to Figure 6A) and **G.** cholesterol biosynthesis (related to Figure 6C). Proteins below the significant value (log2FC >0.59 or <-0.59) are shown in a lighter shade.

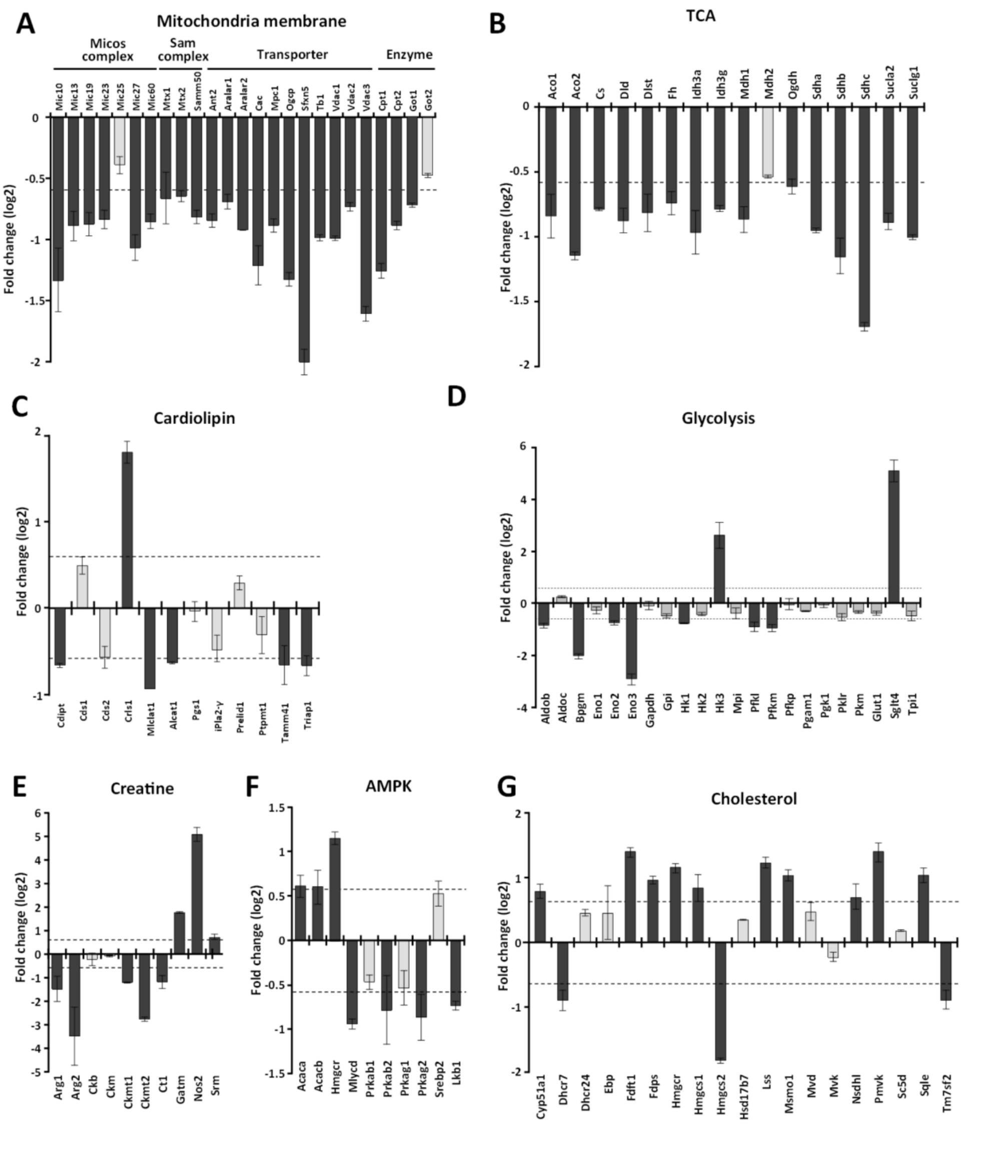

Figure S1

Figure S2. ***C. rodentium*** **impacts β-oxidation (related to Figure 2A).**

Schematic representation of the β-oxidation cycle, with the affected proteins during infection (related to Figure 2A). The bar plot shows the relative abundances of the individual proteins. Proteins below the significant value (log2FC >0.59 or <-0.59) are shown in a lighter shade.

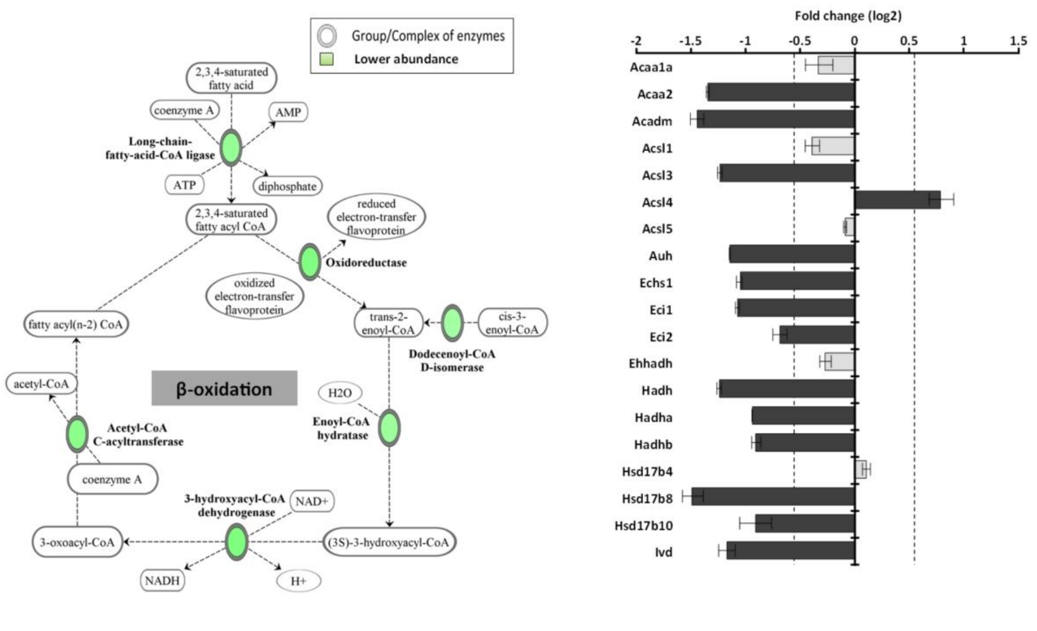

Figure S2

Figure S3. ***C. rodentium*** **impacts lipid elongation (related to Figure 3B).**

Schematic representation of lipid elongation and phosphatidic acid production, with the affected proteins during infection (related to Figure 3B). The bar plot shows the relative abundances of the individual enzymes. Proteins below the significant value (log2FC>0.59 or <-0.59) are shown in a lighter shade.

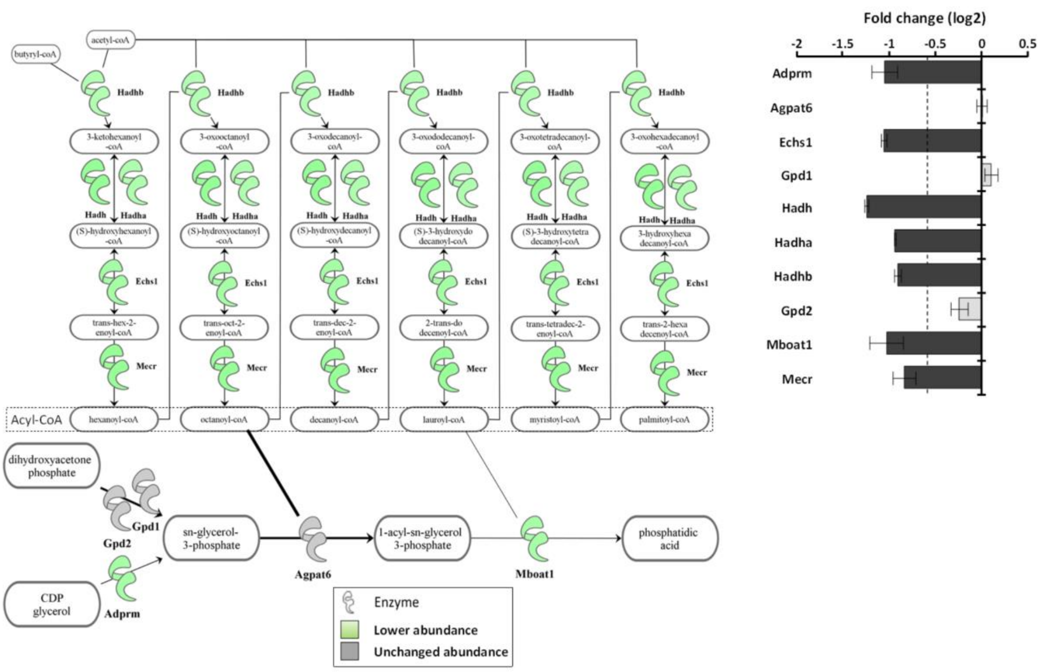

Figure S3

Figure S4. Lipid profile of *C. rodentium* (related to Figure 3B).

A. MALDI-TOF negative ion mass spectra of *C. rodentium*. The absolute abundance of the ions is shown on the y axis, and the masses of the ions are shown on the x axis. The m/z represents mass to charge ratio. **B.** Relative abundance of phosphatidylinositol detected in uninfected and infected IECs. Mann-Whitney test with p-value < 0.05. Each dot represents an individual mouse and bars the geometrical mean. Both panels are related to Figure 3B.

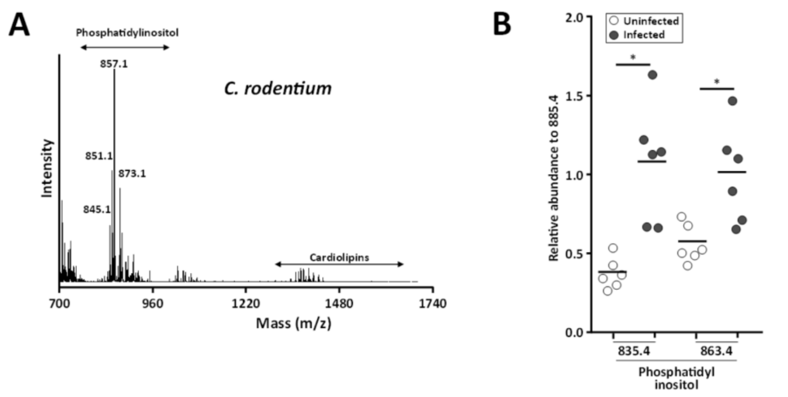

Figure S4

Figure S5. Alpha diversity of tissue-associated microbiota on day 8 p.i (related to Figure 7). Each dot represents and individual mouse and bars the mean.

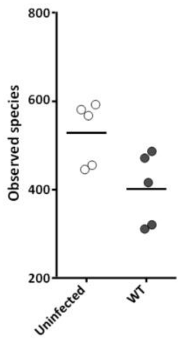

Figure S5

Table S1. List of strains (related to EXPERIMENTAL MODEL)

Strains	Characteristics	Resistance	Source
C. rodentium ICC169	Wild type *C. rodentium*	Nal^R	Schauer and Falkow, 1993
C. rodentium ICC180	*C. rodentium* ICC169::Lux	Nal^R/Kan^R	Wiles et al., 2004
C. rodentium ICC1411	*C. rodentium* ICC180*Δmap*	Nal^R/Kan^R	This study
C. rodentium ICC1412	*C. rodentium* ICC180*Δmap::map*	Nal^R/Kan^R	This study
E. coli CC118λpir	λpir lysogen of *E. coli* CC118	-	Herrero et al., 1990
E. coli CC1047	Wild type helper *E. coli* strain	-	Kaniga et al., 1991

Table S2. List of plasmid (related to Generation of *C. rodentium map* mutant)

Plasmids	Characteristics	Resistance	Source
pSEVA612S-*map*	pSEVA612S containing *map* plus 300bp upstream and downstream	Gm^R	pICC2536
pSEVA612S-*HR*	pSEVA612S containing 300bp upstream and downstream of *map,* without *map* itself	Gm^R	pICC2537
pACBSR	Plasmid containing the endonuclease I-SceI	Sm^R	Ruano-Gallego et al., 2015
pRK2013	Self-transmissible plasmid able to promote conjugal transfer of DNA	Kn^R	Figurski et al., 1979

Table S3. List of primers (related to Generation of *C. rodentium map* mutant)

Primer	Direction	Sequence 5'-3'	Enzyme	Template	Amplicon	Vector
DC074	Forward	TACTGCATGC TGT GCA AGA TCT GTG AGA AAT TGT TCA TTC AT	SphI	gDNA Map	Map + 300bp upstream and downstream	pSEVA
DC075	Reverse	TACTGAGCTC TTT ATA TTG TTA TGA TGC AAC GGT ATG CAG TC	SacI	gDNA Map	Map + 300bp upstream and downstream	pSEVA
DC084	Forward	ATAGAAAAAACATACC AAGCATTTCTCGGT	-	Conjugation colony	Map + 500bp upstream and downstream	-
DC085	Reverse	CAGGGGAGAAAATAAT AAACGAGATCC	-	Conjugation colony	Map + 500bp upstream and downstream	-
